# Microneedle Technology for Overcoming Biological Barriers: Advancing Biomacromolecular Delivery and Therapeutic Applications in Major Diseases

**DOI:** 10.34133/research.0879

**Published:** 2025-11-12

**Authors:** Weishi Ye, Wentao Wu, Siyuan Peng, Zeshi Jiang, Wenhao Wang, Guanlin Wang, Beibei Yang, Fan jia, Anqi Lu, Chao Lu, Chuanbin Wu, Xin Pan, Tingting Peng

**Affiliations:** ^1^ School of Pharmaceutical Sciences, Sun Yat-sen University, Guangzhou 510006, China.; ^2^ State Key Laboratory of Bioactive Molecules and Druggability Assessment, Guangdong Basic Research Center of Excellence for Natural Bioactive Molecules and Discovery of Innovative Drugs, College of Pharmacy, Jinan University, Guangzhou 511436, China.; ^3^ Jiangmen Wuyi Hospital of Traditional Chinese Medicine, Affiliated Jiangmen Traditional Chinese Medicine Hospital of Jinan University, Jiangmen 529031, Guangdong, China.

## Abstract

Biomacromolecules are now widely employed in the therapy of major diseases such as tumors, infections, cardiovascular diseases, metabolic diseases, and autoimmune diseases. Although significant advances have been made in their application, their administration is largely dependent on professional injection, which causes pain, massive medical waste, and high medical burden. As a novel delivery system, microneedles pierce the stratum corneum to assist in intradermal drug delivery, exhibiting minimal invasiveness, high delivery efficiency, and improved patient compliance. The evolution of biomaterials and tissue engineering has broadened microneedle utilization to encompass oral and cardiovascular therapies. Herein, we discuss research advancements regarding the development of biomacromolecule-laden microneedles for treating major diseases. We present detailed examples on how to devise functional microneedles to cross drug delivery barriers in the skin, heart, and blood vessels for improved therapeutic outcomes. Furthermore, we discuss the critical barriers in industrial-scale fabrication and transitioning into clinical practice of microneedles, aiming to provide insightful solutions. Moreover, we explore the future applications of microneedles, including the development of microneedle tacks, robots, and biomimetics to combat intractable diseases. We believe that this review will aid in the designing of microneedles for biomacromolecule delivery, opening up a new avenue for the treatment of major diseases.

## Introduction

In recent decades, the global pharmaceutical market has undergone rapid growth, with a special focus on biomacromolecules. Compared with small molecules, biomacromolecules represented by peptides, antibodies, and nucleic acids are distinguished by their low dosage, high efficacy, and minimal side effects [1]. Biomacromolecules provide distinct advantages over small molecules in the treatment of major disorders such as tumors, infectious diseases, cardiovascular diseases (CVDs), metabolic disorders, and autoimmune diseases. In 2024, biomacromolecules accounted for ≥40% of the new oncology drugs approved by the U.S. Food and Drug Administration [2]. Furthermore, the global pharmaceutical landscape has witnessed remarkable commercial success in biomacromolecules, as evidenced by market leaders like pembrolizumab attaining global sales exceeding $25 billion. This economic impact has made biomacromolecule development a central focus in drug innovation.

**Fig. 1.**
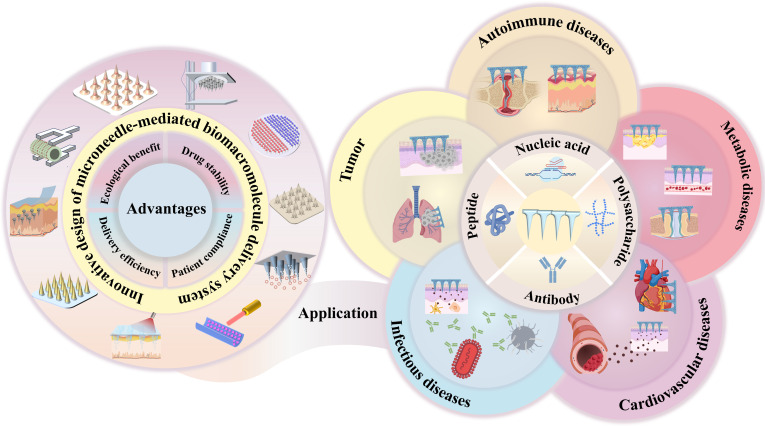
Advantages of microneedle-mediated delivery of biomacromolecules and its application for the treatment of major diseases.

However, to date, the practical application of biomacromolecules is hampered by major challenges, due to their instability and limited bioavailability through traditional administration routes [3,4]. It has become imperative to develop advanced therapeutic transport platforms capable of improving physicochemical stability and patient compliance. The invention of microneedles has opened up a new avenue of painless administration of biomacromolecules. Microneedles mainly comprise micrometer-sized (25 to 1,000 μm) needle arrays and a baseplate, which demonstrates marked superiority compared to traditional percutaneous administration and parenteral injection when facilitating the transport of biomacromolecules (Fig. 1) [5]. For example, (a) they directly pierce the outermost epidermal layer, generating microchannels that enable cutaneous medication administration, demonstrating substantially higher drug delivery efficiency than conventional transdermal preparations such as dermal patches and creams; (b) during the administration process, they do not stimulate dermal vessels and pain receptors, making it painless and popular among patients; (c) microneedles can be self-administered without reliance on medical professionals, and they do not require specific medical waste disposal protocols to be followed, making them particularly suitable for undeveloped areas; and (d) the biomacromolecules loaded in microneedles exist in a solid state and are highly stable, thus reducing the overall costs along the production–distribution–use continuum and providing considerable ecological benefits. As such, the development of microneedles for biomacromolecule delivery is believed to address the bottleneck of injections, holding important potential in clinical practice.

Recent advancements in tissue engineering and biomaterials have substantial broadened the therapeutic scope of microneedles; minimally invasive devices are now being adapted for use in diverse anatomical regions including the gastrointestinal tract, oral cavity, and cardiovascular tissue [6–8]. When applied to the oral cavity, microneedles break through the oral mucosal barriers and deliver the loaded therapeutics to the mucosal tissue, thereby avoiding the cleaning or diluting of the medications by salivary flux. This enables microneedles to provide distinct advantages over conventional oral patches and films, leading to the application of microneedles in oral vaccination, local anesthesia, oral ulcers, and oral tumors [9]. In case of CVDs, microneedles can be customized into implantable medical devices such as microneedle-assisted bypass grafts, endovascular drug-eluting stents, and perivascular drug delivery cuff/meshes to facilitate better distribution and penetration of drugs, thereby increasing the drug delivery efficiency in the disabled heart or blood vessels [10]. Thus, the emergence of intelligent microneedle devices will enable the delivery of drugs to remote inaccessible diseased organs and tissues in the future [11].

Over the past decade, microneedle-mediated delivery of biomacromolecules has emerged as a focal point for global regulatory bodies and multinational pharmaceutical innovators. Biomacromolecule-loaded microneedle products have achieved considerable clinical translation progress, such as insulin-loaded microneedle patches originating from Emory University currently undergoing late-stage clinical evaluation (Phase III) [12]. However, current scholarly efforts have not yet produced a comprehensive synthesis encapsulating cutting-edge innovations in microneedle-facilitated administration of biomacromolecules. In this article, we focus on how to develop biomacromolecule-laden microneedles for the treatment of major disorders such as tumors, infectious diseases, CVDs, metabolic diseases, and autoimmune diseases. We also presented detailed examples on how to devise microneedles with a unique structure or function to cross the drug delivery transport barriers for improved therapeutic outcomes. Despite the considerable advancements, the practical application and clinical translation of microneedles remain constrained by bottlenecks such as low drug-loading and industrial manufacture. We further analyze the potential strategies to address the challenges concerning microneedles and explore the future applications of microneedles. This article is expected to provide critical insights supporting the effective implementation and clinical adoption of biomacromolecules delivered via microneedles. Moreover, it will facilitate the integration of microneedle technology with biopharmaceutical advancements, potentially resulting in major breakthroughs in the treatment of major diseases.

## Application in Infectious Diseases Treatment

Infectious illnesses stem from pathogenic microorganisms including bacteria, viruses, and parasites, posing substantial risks to public health worldwide. Immunization through vaccines represents one of the most impactful and budget-friendly methods to curb and mitigate the spread of pathogens [13]. However, traditional subcutaneous vaccine administration is often associated with challenges such as (a) the requirement of professional personnel for vaccination administration, which can cause pain and poor patient compliance; (b) poor vaccine stability and the high cost of transportation and storage, which is economically unfavorable; and (c) the production of medical wastes such as injection needles, which are not environment friendly [14–16].

Emerging research trends indicate that microneedle transdermal vaccination demonstrates comparative advantages over traditional vaccination [17]. Microneedles can directly transport vaccines through cutaneous layers into the epidermal and dermal strata, which are densely populated with specialized antigen-presenting cells (APCs). The APCs in the skin can induce precise and potent cellular and humoral immune responses even with lower doses of vaccine [18]. Microneedle percutaneous vaccination has been extensively applied for managing and curbing communicable illnesses, emerging as an investigative focus in the biomedical field in recent years.

### DNA vaccine

Nucleic acid vaccines can target selected antigens in the pathogen and transmit its genetic information to recipient organisms; this information directs the biosynthesis of immunologically relevant polypeptides. They have the potential to be safe, effective, and cost-effective [19]. Nevertheless, the limited immunogenicity of nucleic acid vaccines is also a major drawback for their clinical application and translation. Numerous studies have proved that microneedle vaccination can concentrate vaccine components within the dermal microenvironment, reducing the vaccine dosage required for effective immunity, thereby reducing costs and toxicity. For instance, Kim et al. [20] used recombinant DNA technology to design a trimeric subunit vaccine against SARS-CoV-2 S1 and loaded it onto carboxymethyl cellulose-based microneedles. They found that mice exhibited a significant antigen-specific antibody response within 2 weeks postvaccination, with neutralizing antibody titers surpassing those induced by intramuscular injections. These results demonstrated the potent immunogenicity of percutaneous microneedle vaccination. Kuwentrai et al. [21] also developed dissolvable microneedles containing the receptor-binding domain of SARS-CoV-2 spike protein. They similarly found that it elicited strong humoral immunity (B-cell-derived antibodies) and cellular immunity (interferon-γ secreting T cells) in murine models, and maintained the activity for at least 97 days after administration. Furthermore, Wang et al. [22] constructed a universal DNA vaccine against seasonal influenza and delivered it via hyaluronic acid (HA)-based dissolving microneedles. They found that in a seasonal influenza mouse model, the survival rate, antibody titer, T cell response, and germinal center responses of the microneedle group were markedly higher than traditional intramuscular immunization. The findings demonstrate that the microneedle-mediated DNA nanoparticle vaccine can elicit stronger cellular immunity, broader antibody-mediated immunity, and enhanced protection compared to intramuscular injection.

The intrinsic instability of nucleic acid vaccines prevents their long-term storage at room temperature and limits their widespread application. Studies have demonstrated that the combination of microneedles and nanotechnology can remarkably improve the stability of nucleic acid vaccines, thereby prolonging their shelf life under ambient storage conditions. For instance, Yin et al. [23] developed a DNA nanoparticle vaccine-loaded microneedle targeting the protein of SARS-CoV-2. They used the conjugation of deoxycholic acid with low-molecular-weight polyethyleneimine to form nanoparticles that can electrostatically adsorb DNA vaccines. This microneedle improved the stability of the vaccine while achieving effective immunization. Specifically, this DNA-based nanovaccine maintains its immunogenic properties when kept at ambient temperature for up to 1 month. It can provoke a substantial rise in virus-specific immunoglobulin G (IgG) antibody levels postvaccination. Li et al. [24] fabricated a dissolving microneedle incorporating chitosan oligosaccharides and DNA self-assembled nanoparticle vaccine designed to recognize SARS-CoV-2’s spike and nucleocapsid protein. They confirmed that this DNA nanoparticle vaccine loaded onto microneedles remained immunogenic after storage at ambient conditions for over 30 days.

### RNA vaccine

Notably, mRNA vaccines can harness the power of mRNA to evoke robust immune responses against specific antigens. Moreover, mRNA vaccines are much safer than DNA vaccines. They function entirely within the cytosol, circumventing the risk of integration with the host genome. In the global health crisis triggered by SARS-CoV-2, the rapid authorization and commercialization of mRNA-based immunizations developed by Moderna and Pfizer demonstrated the broad prospects of mRNA vaccines [25]. Nonetheless, similar to DNA vaccines, low temperatures are a prerequisite for maintaining the high immunogenicity of mRNA vaccines. Researchers have made considerable progress in addressing their thermostability challenges. Cryogenic microneedles, as a novel microneedle technology, provide a low-temperature environment for pharmaceuticals, thereby improving the stability of mRNA vaccines. Yu et al. [26] designed a cryogenic microneedle incorporating the Comirnaty mRNA vaccine. They found that the cryogenic microneedle-treated group demonstrated robust immune efficacy in countering the original SARS-CoV-2 pseudoviral strain, with over half of replication being suppressed.

### Protein vaccine

With the development of genetic engineering technology, recombinant protein vaccines have emerged as the second-generation vaccines following inactivated and live-attenuated vaccines. These vaccines are produced by integrating pathogen-specific immunogenic protein genes into genetically engineered cell factories (e.g., yeast, *Escherichia coli*, and other microorganisms) [27,28]. Recombinant protein vaccines are composed of pathogenic components (e.g., surface proteins and polysaccharides) and immune-boosting adjuvants. Unlike live vaccines, recombinant protein vaccines cannot replicate in vivo, which eliminates the risk of pathogenicity, thereby demonstrating high safety and stability.

Microneedles can serve as a potent platform for preventive vaccinations or co-administration of vaccines, due to their capacity to deposit vaccines gradually and precisely into the dermis. For instance, Boopathy et al. [29] designed a noninvasive microneedle-based system to administer HIV vaccines. The vaccine components, including the HIV envelope trimer immunogen and an adjuvant, were encapsulated within silk fibroin to form the microneedle’s tip structure. Five minutes after it is applied to the skin, the vaccine-containing silk tips embedded in the skin released the immunogen sustainably as the protein hydrolyzed, with the release rate linked to the crystallinity of the silk fibroin. The immunogen was released for over 2 weeks in mouse models. Compared with single-injection immunization, the serum IgG titer increased approximately 1,300-fold, and the number of bone marrow plasma cells increased 16-fold. Furthermore, Yenkoidiok-Douti et al. [30] designed a gelatin-based dissolving microneedle for delivering a malaria transmission-blocking vaccine. This system incorporated the P47 antigen from *Plasmodium falciparum* along with the Toll-like receptor (TLR)-activating cytosine-phosphate-guanine (CpG) oligodeoxynucleotide. Quantitative enzyme-linked immunosorbent assay revealed that the P47 released from the microneedles exhibited equivalent reactivity to the P47 protein solution at the same dose. Furthermore, flow cytometry demonstrated that splenocytes obtained from immunized mice expressed high levels of CD40, CD80, and CD86 markers. These data indicate that the microneedles can effectively release the P47 vaccine and elicit stronger immune responses than the P47 protein solution.

Numerous studies have demonstrated that the humoral immune responses elicited by repeated immunizations, via either injections or microosmotic pump implantation, are more robust than those elicited by single-shot vaccinations. However, for preventive vaccination, implementing repeated injections is challenging due to the associated pain, high costs, and complex schedules, which impose substantial economic and emotional burdens. To tackle these challenges, Jeong et al. [31] designed a dual-compartment microneedle for atrioventricular administration, incorporating both influenza B antigens (B/Yamagata protein and B/Victoria protein) into separate sections. This design enables dual-antigen delivery via one injection, combining both components into a unified formulation, thereby addressing the complexity of combined vaccines and improving compliance with multiple vaccination schedules. The compartmental microneedle elicited stronger neutralizing antibody levels than those induced by intramuscular injection. Furthermore, the compartmental microneedle exhibited comparable efficacy to that of the 2 vaccinations with single-antigen microneedles. These findings demonstrate that the compartmental microneedle can achieve the effects of multiple vaccinations with a combined vaccine in a single immunization, superior to the immune effects of intramuscular injection.

**Fig. 2.**
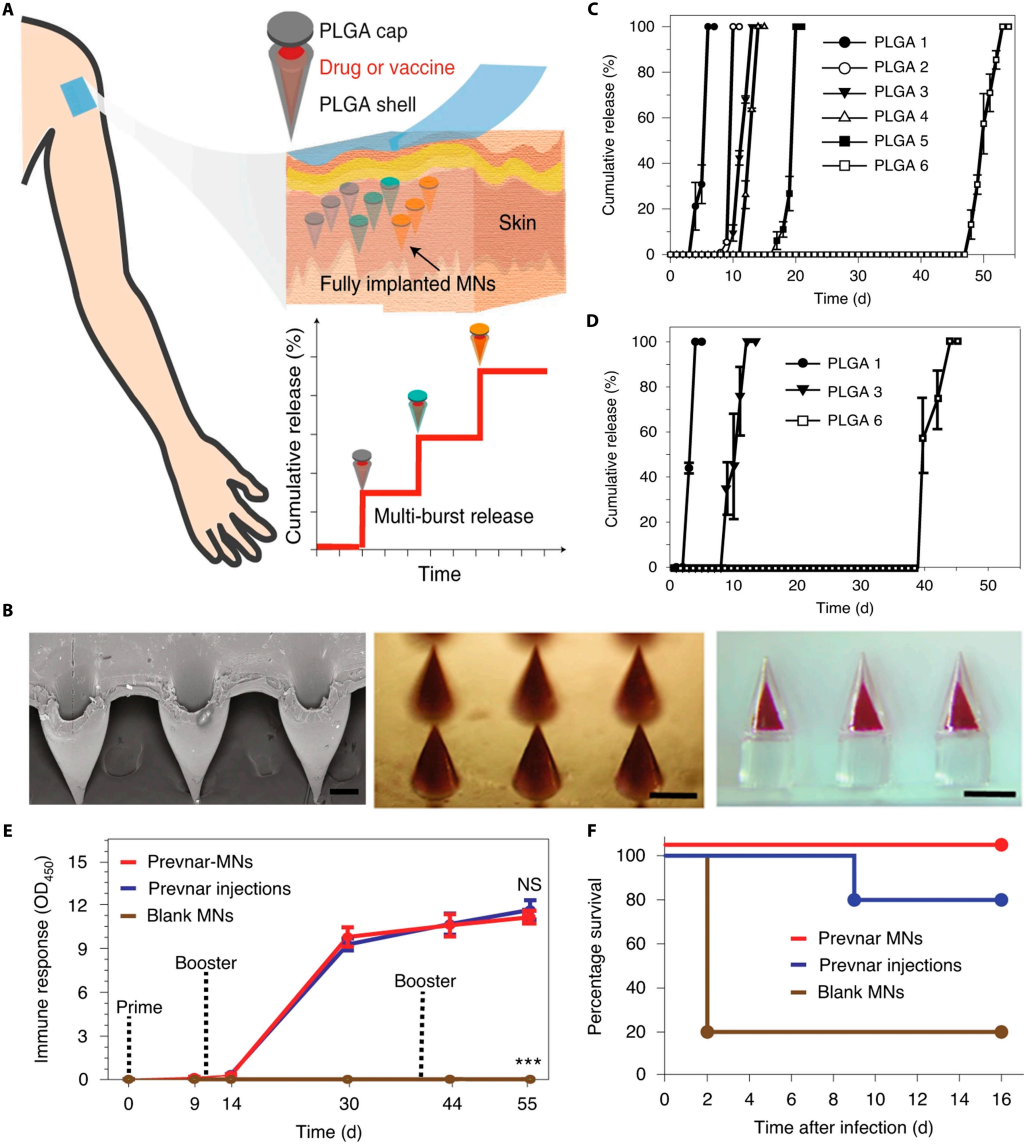
Composition, characterization, and pharmacodynamic evaluation of the programmable burst-release microneedle. (A) Schematic of the programmable burst-release microneedle. (B) Optical image of programmable microneedle. (C) The in vitro release of the microneedle fabricated using various PLGAs (PLGA 1: 1:1 mixture of 15 kDa and 30 kDa acid-endcapped PLGA 50:50; PLGA 2: 1:3 blend of the same 15 kDa and 30 kDa acid-terminated PLGA 50:50; PLGA 3: 30 kDa acid-endcapped PLGA 50:50; PLGA 4: 45 kDa acid-terminated PLGA 50:50; PLGA 5: 60 kDa ester-endcapped PLGA 50:50; PLGA 6: 85 kDa acid-terminated PLGA 75:25). (D) In vivo cumulative release of the microneedle. (E) The immune response of rats administered with Prevnar-13 encapsulated microneedles. (F) The death rates observed in rat models following immunization with Prevnar-13 encapsulated microneedles. These figures were reproduced from Ref. [33] with permission.

Schepens et al. [32] used alumina-based ceramic nanoporous microneedles to deliver recombinant influenza pH1N1 virus hemagglutinin protein vaccines. The protein vaccines diffuse from the nanopores for release. Mice vaccinated via these microneedles showed elevated levels of antigen-specific antibodies compared with those induced by intramuscular injection and were protected against lethal doses of the virus. Tran et al. [33] designed a programmable burst-release core–shell microneedle manufactured by 3-dimensional (3D) technology, which can trigger an immune response similar to that induced by multiple antigen injections. It is composed of the following 3 components: the microneedle shell, the microneedle cap, and the vaccine core (Fig. 2A and B). The microneedles were fabricated from biodegradable poly(lactic-co-glycolic acid) (PLGA), and the breakdown speed of the PLGA shell could be modified to precisely control the release kinetics of the vaccine. Consequently, a single administration of microneedles with multiple PLGA shells resulted in a staggered, burst release of the vaccine over various time intervals, thereby emulating the effects of multiple injections (Fig. 2C and D). The core–shell microneedles loaded with the pneumococcal vaccine induced a strong immune response in rats, exhibiting no significant difference compared with that induced by 3 subcutaneous injections of the vaccine. After receiving lethal doses of pneumococcal attack, all subjects treated with the core–shell microneedle survived, surpassing the 80% survival observed in the injection group (Fig. 2E and F). These data demonstrate that microneedle-mediated vaccination helps to improve the immune efficacy of the vaccine along with reducing the number of vaccinations.

Despite these compelling laboratory achievements, the translation of microneedle-based vaccine delivery into industrial-scale production faces multifaceted challenges. Firstly, academically optimized formulations often struggle with batch-to-batch consistency during mass manufacturing, particularly for genetically engineered vaccines requiring precise nanoparticle assembly. Besides, sterilization remains a critical hurdle, as conventional gamma irradiation or autoclaving may compromise the structural integrity of bioengineered microneedle tips. Regulatory pathways for novel DNA/RNA-loaded microneedles are yet to be standardized, with lingering concerns about long-term stability under real-world distribution conditions. Moreover, while lyophilized microneedle patches demonstrate theoretical cost–benefit advantages, their economic viability necessitates rigorous evaluation in industrial-scale Good Manufacturing Practice (GMP) facilities. Nevertheless, these academic advancements provide critical insights to bridge the translational gap toward clinical microneedle applications.

## Application in the Treatment of Tumors

Tumors are one of the malignant diseases with the highest mortality worldwide, posing a serious threat to human health [34]. According to an Agency for Research on Cancer report, approximately 20% of individuals may suffer from cancer in their lifetime, and the mortality rate remains extremely high. Moreover, projections suggest that annual cancer diagnoses could reach nearly 25 million by mid-century [35], with a huge demand for clinical treatment [36,37]. Although surgical resection, chemotherapy, and radiotherapy are classic tumor treatment methods, they have limitations such as easy recurrence, substantial side effects, and minimal efficacy in treating metastatic tumors. In recent years, immunotherapy has made substantial advancements and has become the mainstay for cancer treatment [38]. It works by reactivating and sustaining the cancer immunity cycle, thereby reinstating the organism’s natural defense against malignancies [39]. However, immunotherapy efficacy remains constrained by physiological barriers and immunosuppressive tumor microenvironments [40]. As a minimally invasive transdermal platform, microneedle technology presents a promising strategy to circumvent these limitations. Distinct from conventional delivery methods, microneedles demonstrate exceptional loading capacity for diverse biomacromolecules (e.g., proteins, peptides, and nucleic acids) while enabling precise spatial delivery to target tissues. This targeted approach minimizes systemic exposure, thereby reducing off-target effects and metabolic degradation. Furthermore, microneedles enhance intratumoral drug distribution and retention through their unique penetration mechanisms. These advantages collectively position microneedles as a rising star in the field of cancer immunotherapy.

### Immune checkpoint inhibitor

As a protective protein in human immune system, immune checkpoint prevents inflammation and damage caused by excessive activation of T cells. Nevertheless, its high expression in the tumor microenvironment of several malignant tumors impedes the efficacy of immune-based treatments [41]. Consequently, immune checkpoint inhibitors (ICIs) are effective tumor immunotherapies since they reactivate the immune response effect of T cells on tumors [42]. Common ICIs include programmed death receptor-1 (PD-1) inhibitors and cytotoxic T lymphocyte-associated antigen 4 (CTLA4) inhibitors.

The most common ICIs are PD-1 inhibitors. Although it has been successfully applied in clinical practice, there are flaws in the current treatment process, such as low overall response rate and high incidence of inflammation [43]. Wang et al. [44] designed a dual immune microneedle to load PD-1 inhibitors into microspheres formed by the self-assembly of HA and 1-methyltryptophan (1-MT), an indoleamine 2,3-dioxygenase small-molecule inhibitor, which can up-regulate T cell activity, inducing stronger immune response effects. Compared with a single treatment method, this combination therapy of immune microneedle patches improved the survival probability of mice with tumors by approximately 70%. Based on that, they designed dissolving microneedles that continuously deliver PD-1 inhibitors in a pH-responsive manner, playing a physiological regulatory role in release. The principle relies on the gradual self-disintegration of glucan nanoparticles in the acidic environment of cancerous tissues, leading to the controlled liberation of PD-1 inhibitors. The immune response in the microneedle group significantly improved, and the percentage of cluster of differentiation CD8^+^ T cells was 3 times that in the free treatment group. Furthermore, the microneedle group could continuously release the encapsulated drug within 3 days of administration, exhibiting a sustained release effect [45]. To improve the drug-loading capacity, Yang et al. [46] designed a microneedle with a core–shell structure containing a high drug load, employing polyvinyl alcohol (PVA) as the needle core due to its abundance of both hydrogen bond-donating and -accepting groups. Through hydrogen bonding between PVA and 1-MT, the early-stage crystallization of 1-MT is suppressed, leading to improved drug incorporation efficiency. The charged shell prepared from chitosan was used to concentrate anti-PD-1/L1 antibodies through electrostatic interactions. In the C57BL/6 mouse melanoma model, the microneedle group demonstrated prolonged drug persistence in the target area, leading to a significantly increased accumulation of T cells within the tumor site. Moreover, the antitumor efficacy was superior to that of the intertumoral injection group, indicating that microneedles could function as a promising and effective system for delivering tumor ICIs.

Beyond PD-1 inhibitors, microneedle systems loaded with hydrophilic/hydrophobic CTLA4 inhibitors have been reported. Chen et al. [47] used modified dextran with pH-responsive capabilities and hydrophilic aCTLA-4, along with the hydrophobic photosensitizer zinc phthalocyanine (ZnPc), to self-assemble into nanoparticles for constructing an intelligent responsive dissolving microneedle transdermal delivery system for controlled drug release. Under near-infrared (NIR) light irradiation, photodynamic therapy kills tumor cells and produces large amounts of related antigens, which together with ICIs trigger a dual immune response. Compared with that in the blank microneedle group, the infiltration of CD3^+^ T cells, CD8^+^ T cells, and CD4^+^ T cells significantly increased in the pH-responsive microneedle group, with a noticeable reduction in tumor volume.

**Fig. 3.**
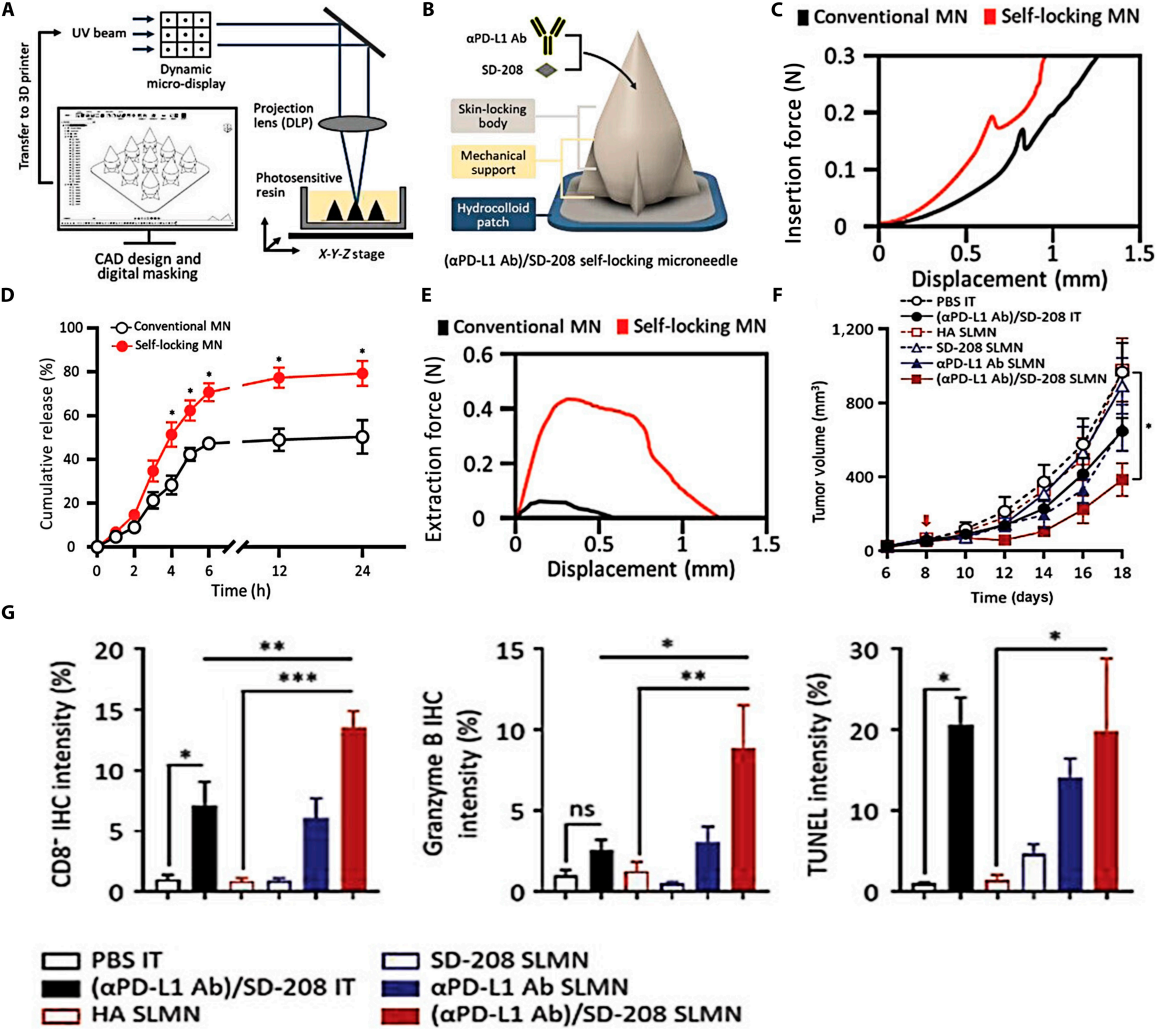
Composition, characterization, and pharmacodynamic evaluation of SLMN. (A) Manufacturing methodology of SLMN. (B) Schematic illustration of SLMN. (C) Skin insertion force of microneedles. (D) The kinetic analysis of dermal penetration for SLMN and conventional microneedles. (E) Skin extraction forces of microneedles. (F) Growth patterns of tumor in differentially treated mice [PBS IT: Direct injection of PBS; (αPD-L1 Ab)/SD-208 IT: Direct injection of αPD-L1 Ab/SD-208; HA SLMNs: blank MNs; SD-208 SLMNs: SD-208-loaded MNs; αPD-L1 Ab SLMNs: αPD-L1 Ab-loaded MNs; (αPD-L1 Ab)/SD-208 SLMNs: (αPD-L1 Ab)/SD-208-loaded MNs]. (G) The positive ratio of CD8-IHC, Granzyme B IHC, and TUNEL obtained from immunofluorescent staining images. These figures were reproduced from Ref. [48] with permission.

The above-mentioned reports collectively demonstrate that constructing intelligent responsive systems for microneedles to exert a sustained-release effect can improve the dose effect of drug delivery. Nevertheless, the natural elasticity of cutaneous tissues can restrict the effectiveness and precision of traditional microneedle systems in drug administration. Therefore, developing more ingenious secondary structures of microneedles is an important direction for improving immunotherapeutic outcomes. Joo et al. [48] used additive manufacturing techniques to create a microneedle system (SLMN) for the codelivery of PD-L1 antibodies and SD-208 (a TGF-β inhibitor) (Fig. 3A). It consisted of a tapered apex for epidermal penetration, an expanded midsection to anchor within the dermal layers, and a constricted foundation ensuring secure cutaneous retention post-insertion (Fig. 3B). Their results demonstrated higher skin adhesion, drug permeability, and delivery accuracy in the self-locking microneedle groups than in the traditional microneedle groups (Fig. 3C to E). Moreover, in the melanoma mouse model, compared with that in the intratumoral injection group and the blank self-locking microneedle group, the tumor growth volume suppression effect in mice treated with self-locking microneedles maintained continuously for 18 days. As depicted in Fig. 3F, a 14-fold enhancement in CD8 fluorescence signal was observed within the tumor region (Fig. 3G).

### Tumor vaccines

Tumor vaccines improve the competence of the body’s immune system to recognize and kill tumor cells by targeting tumor-associated or tumor-specific antigens, thus exerting substantially therapeutic effects in various solid tumors [49]. Li et al. [50] designed a rapid dissolution microneedle based on polyvinylpyrrolidone (PVP), which included chitosan nanoparticles encapsulated in the model antigen ovalbumin (OVA) and TLR9 agonist for the efficient transdermal delivery of vaccines. Compared with traditional subcutaneous injection, microneedle injection could achieve the same level of immune response in a more convenient and minimally invasive manner.

**Fig. 4.**
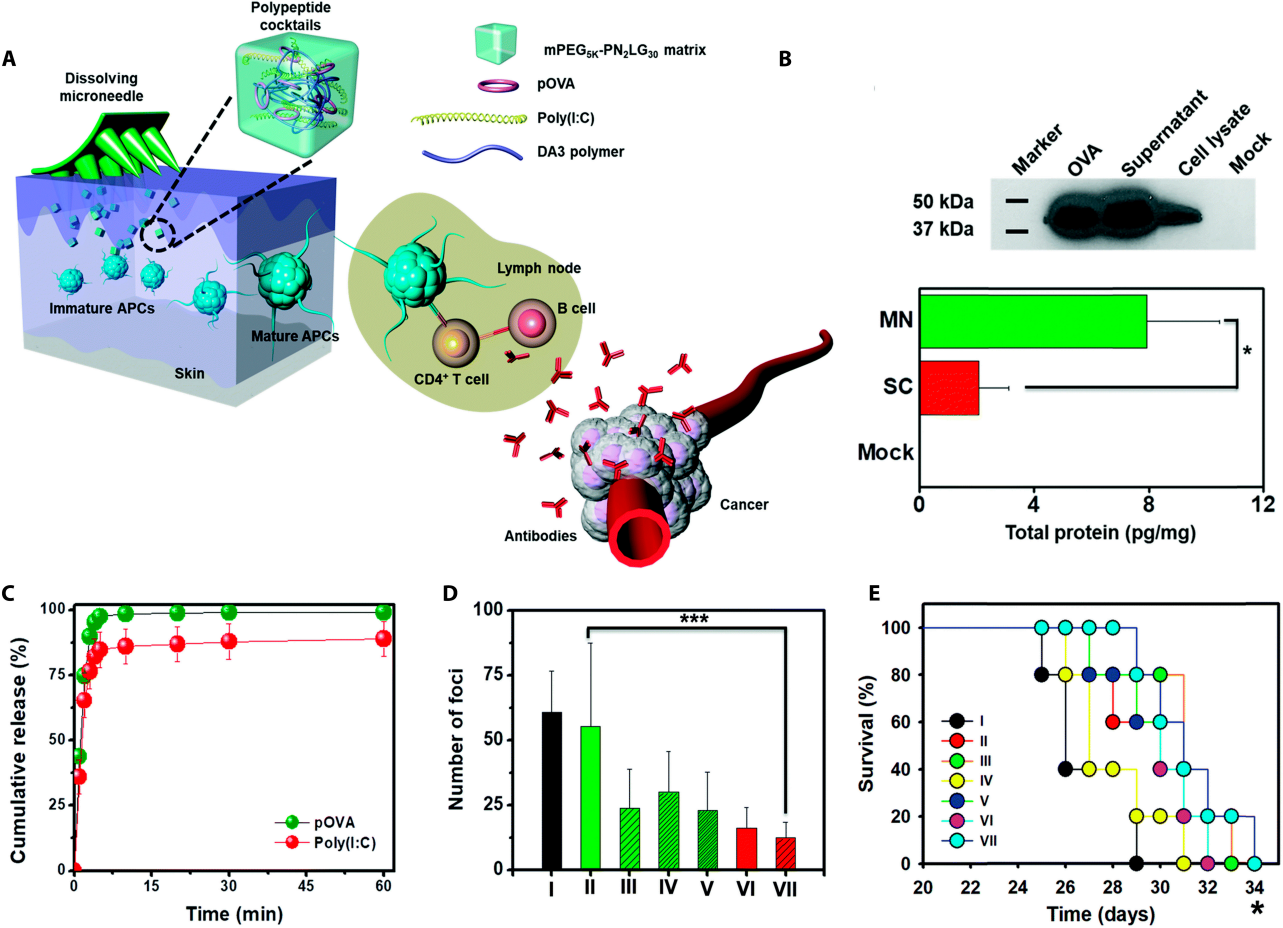
Composition, characterization, and pharmacodynamic evaluation of polypeptide cocktail-loaded dissolving microneedles. (A) Schematic diagram of microneedle-mediated transdermal delivery of poly(I:C) and pOVA. (B) In vivo OVA expression at 72 h post-administration in murine models. (C) In vitro release curves of microneedles loaded with pOVA and poly(I:C). (D) Quantitative assessment of pulmonary lesion foci across different formulation cohorts [I: control group; II: subcutaneous injection of OVA antigen, respectively; III: subcutaneous injection of OVA antigen and poly(I:C), respectively; IV: subcutaneous injection of DA3/pOVA, respectively; V: subcutaneous injection of DA3/pOVA and poly(I:C), respectively; VI: dMN-mediated delivery of DA3/pOVA, respectively; VII: dMN-mediated delivery of DA3/pOVA and poly(I:C), respectively]. (E) Survival percentage of different treatment groups. These figures were reproduced from Ref. [52] with permission.

Nucleic acid vaccines possess the capability to transmit the genetic information of viral antigens to host cells, which enables the host cells to produce corresponding proteins, helping the human body form immune memory to respond promptly. DNA vaccines exhibit low immunogenicity in clinical trials; however, microneedle-mediated intradermal delivery enhances their potency, enabling durable immune protection. As such, Duong et al. [51] designed an intelligently adaptive microneedle for delivering nanoparticles composed of a DA3 cationic copolymer and ovalbumin granules (pOVA). The microneedle consisted of a negatively charged poly (I:C) and a positively charged polymer (OSM-[PEG-PAEU]) assembled layer-by-layer (LbL) on the microneedle surface. The responsive principle is that OSM-(PEG-PAEU) turns into an anionic copolymer at physiological pH, causing the LbL layers to disintegrate due to electrostatic repulsion, leading to the swift liberation of cationic nanoparticles along with poly(I:C). The positive charge of the DA3 copolymer can effectively improve the cellular uptake of nanoparticles, and its amphiphilicity can promote the release of pOVA through the “proton sponge” effect. Poly (I:C) acts as a TLR3 agonist. Once it binds to TLR3 in immune cells, it triggers the activation of B lymphocytes, thereby enhancing antibody-mediated immunity. Compared with subcutaneous injection of DNA vaccine, this intelligent response vaccine microneedle enhanced antibody and cytotoxic CD8^+^ T lymphocyte production by 3 times in mice, indicating higher efficacy, compliance, and safety. On the basis of this research, they further designed a dissolving microneedle for peptide cocktail delivery (Fig. 4A), which consisted of poly(I:C) and pOVA, assembled with DA3 to generate stable nanoparticles [52]. The material of microneedles was a copolymer (mPEG_5K_–PN_2_LG_30_) composed of cationic peptides and polyethylene glycol (PEG), which is biocompatible, bioabsorbable, and hydrophilic. The hydrophilic properties of cationic peptides allowed for a burst release of 97% of the nanoparticles within 5 min (Fig. 4B), and the released pOVA could be effectively captured by APCs (Fig. 4C). In B16F10-OVA melanoma mice with lung metastasis, the microneedle group exhibited significant tumor regression after 14 days of treatment (Fig. 4D), whereas the subcutaneous injection group and OVA antigen group alone showed no significant tumor suppression effects. Moreover, 20% of the animals remained alive at 34 days post-intervention in microneedle-treated cohort, in contrast to the control group that exhibited complete mortality by this time point, indicating that the microneedle-mediated peptide cocktail could effectively improve the efficacy of tumor vaccines (Fig. 4E).

As micrometer-scale devices, microneedles inevitably face the challenge of limited load capacity, considering that sufficient delivery of DNA vaccines is the foundation for initiating immune responses. Cole et al. [53] developed a new manufacturing process that uses freeze-drying technology to improve the loading capacity of microneedles. They combined cationic penetrating peptides with cervical cancer pDNA vaccines, assembling them into nano-sized particles, which were then incorporated into a PVA matrix to fabricate the microneedle system. The resulting dissolving microneedle patches had a loading capacity approximately 7 times higher than that of conventional microneedle patches, and the mechanical strength and vaccine functionality of the microneedles remained unchanged over 28 days without notable alterations. In the cervical cancer mouse model, the tumor weight reduced by 3.6 times in the microneedle group relative to the control group, whereas this reduction was only 2.3 times in the intramuscular injection group, indicating that the system could exert effective therapeutic antitumor effects and was superior to intramuscular injection. van der Maaden et al. [54] designed a numerical control hollow microneedle injection system based on fused silica to deliver cationic liposome HPV E7_43–63_ containing the SLP long peptide. The system could accurately inject a control volume as low as 1 μl, minimized drug loss and pain, and improved the effectiveness of the vaccine. Compared with traditional subcutaneous injection, immunization using microneedles elicited 3 times higher proportion of CD4^+^ T cells.

### Gene therapy

Nucleic acid drugs can directly block the expression of pathogenic genes and possess several therapeutic advantages, including the following: (a) The sequence design of this kind of drug is simple, as it relies solely on Watson–Crick hybridization rules [55]. The discovery process for small molecules and antibody drugs is cumbersome. (b) Small-molecule and antibody drugs have limited target locations [56]. Conversely, nucleic acid drugs can directly regulate genes that express related proteins, bypassing the need to interact with protein targets. This can avoid the limitations associated with traditional drug strategies [57]. The microneedle-mediated local administration of nucleic acid-based therapeutics can improve its accumulation in superficial cutaneous tumors and augment the efficacy of gene therapy, demonstrating immense promise in the field of tumor therapy.

Plasmid DNA, a negatively charged biomacromolecule, can be fabricated into gene-loaded polyelectrolyte multilayer (PEM) membranes through the LbL self-assembly technology. Li et al. [58] developed a polycaprolactone microneedle coated with pH-responsive PEM using the LbL technology, achieving rapid release of p53 expression plasmids in acidic skin environments. The PEM is composed of 2 functional strata: a bridging film incorporating PLL-DMA (poly-L-lysine altered with dimethylmaleic anhydride) and a loading stratum containing the p53 plasmid. Under low pH conditions typical of cutaneous surfaces, the anionic PLL-DMA in the bridging film becomes positively charged, causing the layer to collapse due to charge repulsion and facilitating gene release from the loading layer. In vivo studies demonstrated that the microneedle-based treatment achieved a 90.1% suppression rate of tumor growth in a subcutaneous tumor mouse model, whereas it was only 30.5% with intravenous administration, thus indicating that the microneedle can effectively facilitate p53 plasmid delivery, enabling therapeutic intervention against subcutaneous tumor growth.

**Fig. 5.**
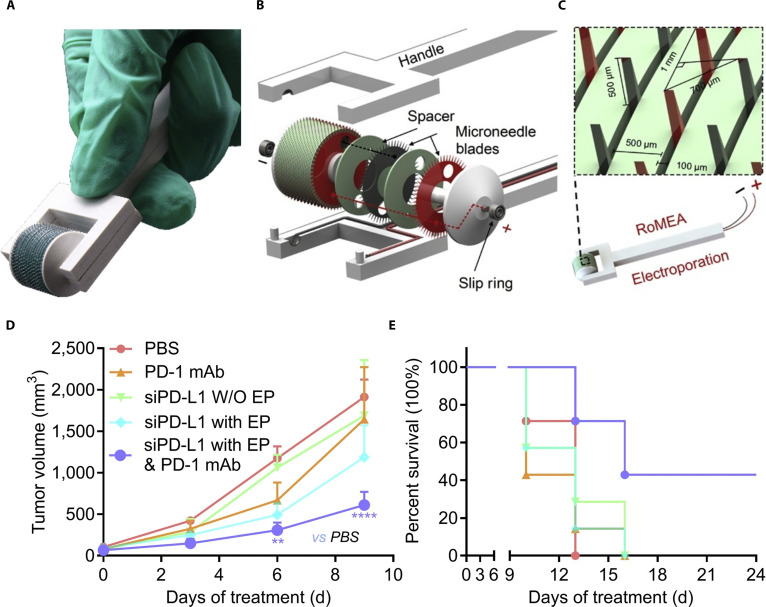
Composition, characterization, and pharmacodynamic evaluation of RoMEA. (A) Schematic of the handheld appearance of the device. (B) The portable design of the device. (C) Demonstration of sustained electroporation for tissue-specific applications via a minimally invasive device. (D) Tumor growth curve of the B16F10 melanoma model mice during treatment (PD-1 mAb: anti-PD-1 monoclonal antibody; siPD-L1 W/O EP: siRNA targeting PD-L1; siPD-L1 with EP: siRNA targeting PD-L1 though RoMEA; siPD-L1 with EP & PD-1 mAb: siRNA targeting PD-L1 and PD-1 mAb though RoMEA). (E) Survival curve of B16F10 melanoma model mice. These figures were reproduced from Ref. [13] with permission.

RNA-based therapeutics are devoid of genotoxicity risks [59]. Small interfering RNA (siRNA) enables selective gene suppression, diminishing the production of disease-causing proteins, thereby making this novel therapy a highly promising and efficient tumor treatment method. Pan et al. [60] developed biocompatible dissolving microneedles loaded with STAT3 siRNA encapsulated in polyacetylimide for melanoma treatment. In the melanoma mouse model, the microneedle group exhibited a significantly lower relative mRNA expression level than the blank control group, with a tumor necrosis area of ~40%. Furthermore, Yang et al. [13] designed a rolling microneedle electrode array (RoMEA) to enable highly effective siRNA therapy in vivo. The device employed concentrically arranged disk-shaped cutters incorporating microneedle arrays along their peripheries to serve as conductive elements. This precise structure enables adequate electrical potential differential across the dermal and muscular layers, ensuring efficient transfection at low voltage (Fig. 5A to C). In vivo experiment showed that the microneedle device codelivered siRNA targeting PD-L1 and the PD-1 inhibitor. Results revealed a significant decrease in the tumor volume, with 80% of mice surviving over 24 days, thereby indicating that this strategy restored immune response and inhibited tumor growth (Fig. 5D and E).

## Application in the Treatment of CVDs

CVDs represent one of the paramount threats to human health [61]. According to the American Heart Association, over 17.3 million people die from CVD annually, and this number will exceed 23.6 million by 2030 [62]. CVDs are a group of disorders related to blood vessels and heart, such as myocardial infarction (MI), thrombosis, and hypertension. Drug intervention and surgery are 2 mainstays for CVD treatment, while they have unavoidable drawbacks of uncertain therapeutic effects, substantial impairments, and poor patient compliance [63]. The emergence of microneedles provides new hope for safe and efficient treatment of CVD, as microneedles can be used as a minimally invasive device to attach to the target tissue for precise treatment. As shown in Table 1, microneedles have emerged as an effective transdermal platform for biomacromolecules delivery such as proteins, nucleic acids, and polysaccharides in CVD therapy in recent research.

**Table 1.** Recent studies on the microneedle-mediated delivery of biomacromolecules for the treatment of cardiovascular diseases

Cardiovascular diseases	Types of biomacromolecules	Microneedle design	Main features	Reference
Myocardial infarction	Protein	Near-infrared light triggered self-folding microneedles made of graphene oxide and VEGF were developed to treat myocardial ischemia	The pre-folded microneedles can rapidly recover their shape and adhere securely to the cardiac tissue within 10 s for drug administration	[70]
	Peptide	A bilayer mucoadhesive microneedle was designed to improve drug retention in myocardia	Compared with medical tape, the microneedle bandage can better adhere to the isolated porcine myocardial tissue and prolong the retention of therapeutic peptides in myocardia	[72]
	Nucleic acid	The AAV phase transition microneedles coated with VEGF gene were developed to achieve uniform drug distribution in myocardium	The viral vector delivered by microneedles can be uniformly distributed and transfected in the myocardium, resulting in significantly higher delivery efficiency than direct injection	[74]
Thrombus	Polysaccharide	A thrombin-responsive anti-thrombotic microneedle made of thrombin cleaving peptide-modified hyaluronic acid was developed to intelligently treat thrombus	Compared with non-responsive microneedles, the thrombin-responsive microneedles can prevent burst release of heparin and protect mice from acute thromboembolism	[83]
	Peptide	Microneedles loaded with recombinant bifunctional hirudin containing RGD sequence were developed to achieve superior anticoagulant effect	The recombinant hirudin-loaded microneedle significantly reduced the overall embolism degree, resulting in comparable antithrombotic effect to subcutaneous injection	[87]

### Myocardial infarction

MI, primarily caused by coronary artery occlusion, is the most common type of CVDs. The occurrence of MI leads to massive heart muscle cell death and excessive inflammatory response, subsequently resulting in fibrotic scarring of the myocardium, structural cardiac alterations, and ultimately progressive cardiac dysfunction with fatal outcomes [64]. Among all CVDs, the global mortality of acute MI ranks first and will even continue until 2030 [65]. Uncontrolled excessive cardiac fibrosis is recognized as the primary factor contributing to mortality following MI. The main treatments for MI include utilizing supportive biological stents such as injectable hydrogels and cardiac patches, as well as delivering antifibrotic drugs or bioactive factors. However, direct intracardiac injection of drugs or intervention with supportive biological scaffolds may lead to structural alterations in the surrounding extracellular matrix and cause undesired inflammatory responses [66]. As an advanced local drug delivery technology, microneedles have become a potential tool for the treatment of MI.

#### Growth factor-based therapy

The repair and regeneration of damaged blood vessels or tissues after MI has attracted more attention. Many novel therapeutic strategies for regenerating damaged blood vessels have been developed. Most focused on the stem cell- or growth factor-based therapy. Stem cell-based therapy belongs to the first generation of therapeutic strategies, using cells derived from different sources to promote myocardial regeneration [67]. However, the majority of the positive outcomes linked to stem cell therapy are largely due to the paracrine influence exerted by the growth factors produced by stem cells [68].

Mesenchymal stromal cells (MSCs) notably contribute to angiogenesis by secreting pro-angiogenic mediators in a paracrine manner, including vascular endothelial growth factor (VEGF), hepatocyte growth factor (HGF), and insulin-like growth factor (IGF). The limited viability of transplanted cells and their inability to localize accurately at target sites hinder the application of MSCs. Recently, Hu et al. [69] designed a suture-free, detachable microneedle to deliver MSC-derived factor (MSCF) for the management of MI. Human bone marrow-derived MSCF was loaded in the PLGA nanoparticles to replicate the secretory functions characteristic of stem cells. MSCF was further loaded in the needle tips, which were fabricated from an elastin-like polypeptide (ELP) hydrogel to promote cell attachment. The water-soluble property of HA permits its application in developing microneedle bases that undergo detachment from the tips upon interaction with pleural effusion. The detachable base can prevent microneedles from attaching to the chest wall, thereby reducing arrhythmias due to the extra pressure caused by the patch. In addition, the ELP gel dissolves almost completely within 28 days, leaving no harmful material residue. In the MI rat model, the needle tip was securely positioned within the cardiac muscle tissue by a miniature suction apparatus, continuously releasing MSCF to reduce local myocardial damage and inflammation. Compared with the needle-free patch group, the MSCF in the microneedle group was released after 3 to 7 days, which could effectively avoid the uptake of monocytes, allowing MSCF to penetrate deeper into the myocardium.

**Fig. 6.**
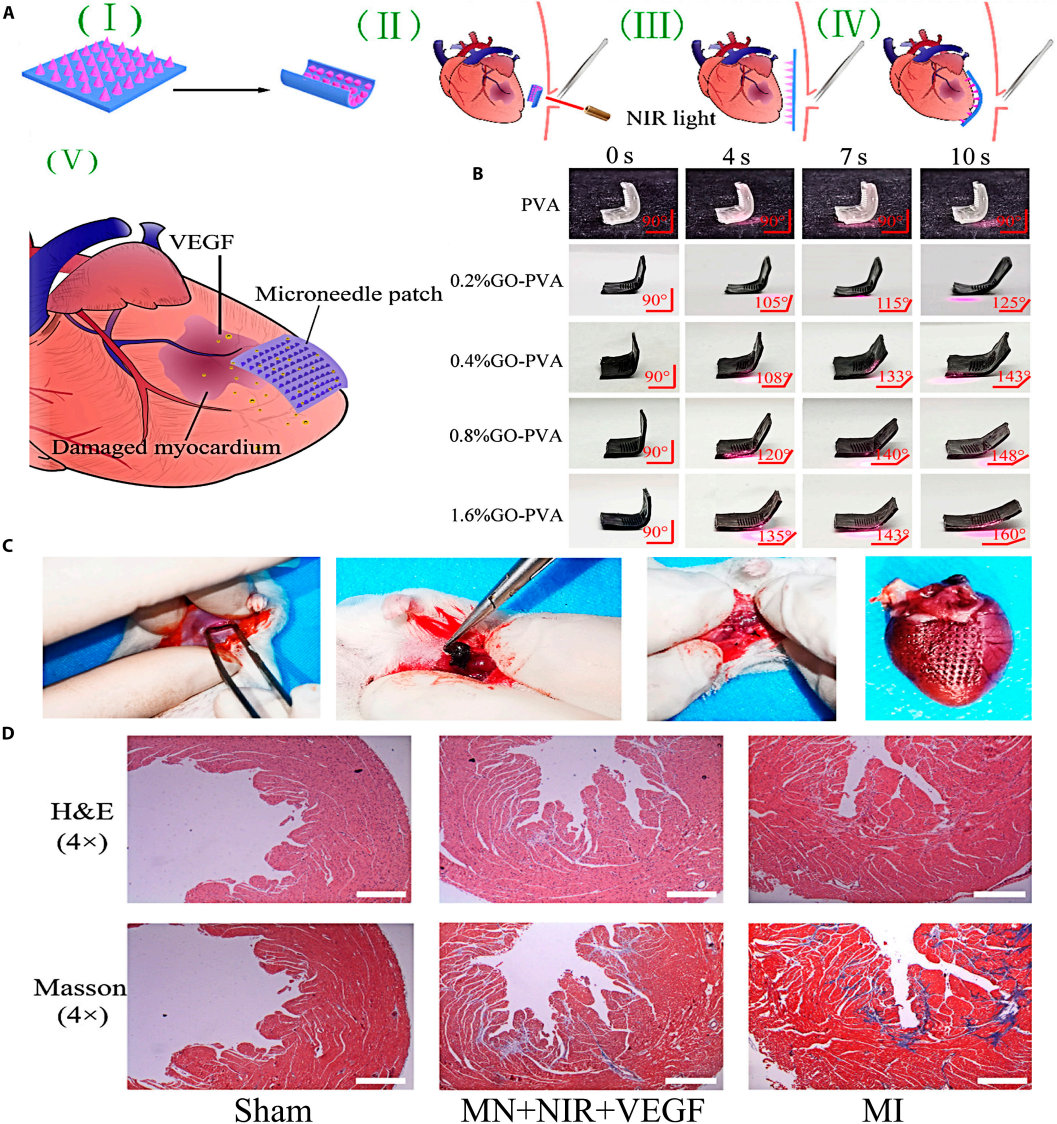
Schematic diagram and pharmacodynamic evaluation of NIR-triggered unfolding microneedle. (A) General framework for MI intervention based on a GO-PVA microneedle. (B) Photographic records capturing the restoration of different GO-PVA hydrogels. (C) Images capturing the sequential phase of the minimally invasive surgery; from left to right are the minimally invasive incision of the chest cavity, the insertion of the pre-folded microneedles into the incision, the suture of the incision, and the microneedle marks left on the removed heart. (D) The changes of the myocardial morphology in the MI heart sections (Sham: sham operation group; MN+NIR+VEGF: NIR-triggered VEGF-loaded microneedle; MI: positive control group). These figures were reproduced from Ref. [70] with permission.

To provide a safe and minimally invasive therapeutic regimen, Fan et al. [70] designed a self-unfolding microneedle activated by NIR, which was made of graphene oxide (GO) and PVA to deliver VEGF for topical treatment of MI. GO has excellent photothermal conversion performance to enable the pre-folded microneedles to unfold within 10 s under 808 nm NIR irradiation (Fig. 6A and B), thereby achieving minimally invasive surgical implantation of the microneedles. In MI mouse models, the application of microneedles required only a thoracic opening of less than 4 mm (Fig. 6C), and the entire procedure could be completed within 1 min. Compared to the negative controls and the empty microneedle-treated subjects, the drug-loaded microneedle subjects that received NIR irradiation exhibited significantly increased neovascularization and reduced myocardial fibrosis. These results indicate that the unfolded microneedles could effectively adhere to the cardiac surface and deliver VEGF to local ischemic myocardium for satisfying therapeutic effects (Fig. 6D).

Although growth factors have been demonstrated to possess good angiogenic activity and improve myocardial perfusion by restoring the structure and function of cardiomyocytes, this therapy failed to yield the anticipated uniform efficacy across patients. Their therapeutic potential in CVDs is constrained by their rapid clearance from the bloodstream and inadequate durability [71]. Lim et al. [72] devised a double-layer adhesive microneedle bandage to topically deliver growth factors to ischemic myocardium for enhanced treatment of MI. The bottom layer of the microneedle bandage was made of a biofunctional protein glue (MAP-BFP) fused with VEGF and mussel adhesion protein (MAP), while the surface layer was made of stiff silk fibroin connected by visible light-activated dityrosine crosslinking. They proposed that this microneedle could serve as an adhesive for tissue repair in moist and mechanically active environments, leveraging the natural wet-adhesion characteristics of biologically engineered MAP combined with the structural reinforcement provided by silk fibroin. Meanwhile, the adhesive microneedles prolonged the retention time of VEGF and MAP and increased the infarct wall thickness by more than 2 times, satisfactorily ameliorating adverse remodeling in MI.

#### Gene therapy

Gene therapy targeting angiogenesis and cardiac regeneration represents a potential treatment option for ischemic myocardial disease. The effectiveness of gene therapy largely depends on the successful transfer of genetic material to myocardium. Although local areas around the injection site can achieve a high density of gene transfer, gene transfection is almost negligible in the areas beyond 5 mm of the needle [73]. To achieve homogeneous gene transfer in the myocardium, Shi et al. [74] developed a microneedle coated with adeno-associated virus (AAV) expressing VEGF gene to treat ischemic myocardial disease. Bioluminescence imaging demonstrated that the viral vector can be uniformly distributed and transfected in the myocardium while myocardial cells transfected with AAV through direct intramyocardial injection (DI) were limited to the injection site. Moreover, the scar and infarct areas observed in the microneedle-treated cohort were markedly reduced in comparison to both the blank control group and the DI group, indicating that microneedle-mediated AAV gene therapy can achieve more efficient and targeted delivery of VEGF to myocardial cells. Compared with systemic administration, topical administration of AAV via microneedles required lower dose and produced lower dose and produced a lower immune risk. Therefore, microneedle provides an alternative option for minimally invasive gene therapy of MI.

MicroRNA (miRNA) is a class of small noncoding RNA that can cause mRNA degradation or translational repression. Accumulating studies have demonstrated that miRNA can improve neovascularization and prevent subsequent negative cardiac remodeling by direct reprogramming of cardiomyocytes [75]. Overexpression of miRNA-30d (miR-30d) was found to prevent cardiomyocyte apoptosis as well as avert cardiac fibroblast proliferation [76]. To achieve local and prolonged delivery of miR-30d to cardiac tissue, Chen et al. [77] designed a conducting microneedle array integrating miR-30d laden with zeolitic imidazolate framework-8 (ZIF-8) nanoparticles and gold nanoparticles. The encapsulation of miR-30d in the ZIF-8 nanoparticles can prevent its degradation in the lysosome, while facilitating its release from lysosomal compartments through the proton-absorbing mechanism. Gold nanoparticles doped on the microneedles can reconstruct the electrical impulses of the infarcted myocardium. In the myocardial injury mouse model, the mouse treated with the conductive microneedle exhibited significant improvements in cardiac function and standard electrocardiogram waveforms. The therapeutic effects of conductive microneedle lasted for up to 6 weeks after implantation, indicating that the microneedle patch can serve as a long-term cardiac device.

Following MI, the expression of miR-29b is significantly reduced, a trend also observed in cardiac hypertrophy models, and this down-regulation may play a role in promoting fibrotic remodeling and scar tissue development [78]. Thus, up-regulating the levels of miR-29b may offer therapeutic advantages in inhibiting fibrotic changes in the myocardium and promoting the regeneration of cardiac tissue. Yuan et al. [79] used microneedles to deliver miRNA-29b (miR-29b) mimics, which possess anti-fibrotic activity, packaged within exosomal vesicles derived from mesenchymal stem cells of human umbilical cord origin. In the MI mouse model, the microneedle treatment group showed a significant reduction in inflammatory responses, collagen deposition, and fibrosis, which generated a considerable therapeutic effect to the sham surgery group. A notable decrease in both the extent of necrotic tissue and collagen deposition was observed compared with MI cohorts. These results indicate that microneedles can significantly enhance exosome retention within the myocardial tissue affected by infarction.

### Thrombosis

Thrombosis is a class of vascular disorders that have been implicated as a potential contributor to the development and progression of CVDs, such as MI and stroke, leading to high mortality and high disability rates [80]. A total of 100,000 to 300,000 people die annually from venous thromboembolism in the United States [81]. Conventional treatment methods primarily rely on anticoagulant agents such as heparin (HP).

HP is the most commonly used anticoagulant and exhibits a molecular mass ranging between 15 and 18 kDa. HP has poor transdermal permeability, owing to its high molecular weight. In the clinic, HP is typically administered through frequent intravenous and subcutaneous injections for thrombosis treatment, because of its substantial first-pass metabolism and short half-life. With the assistance of microneedles, HP can be delivered to systemic circulation via a transdermal administration route. Arshad et al. [82] developed an HP sodium-loaded dissolving microneedle to treat venous thromboembolism. After applying the microneedle to rabbit skin, the activated partial thromboplastin time (APTT) exhibited a marked increase, reaching 160 s, which was 4 times higher than that of the negative control group. This result demonstrates that microneedles incorporating HP exhibit promising therapeutic efficacy against venous thromboembolism.

**Fig. 7.**
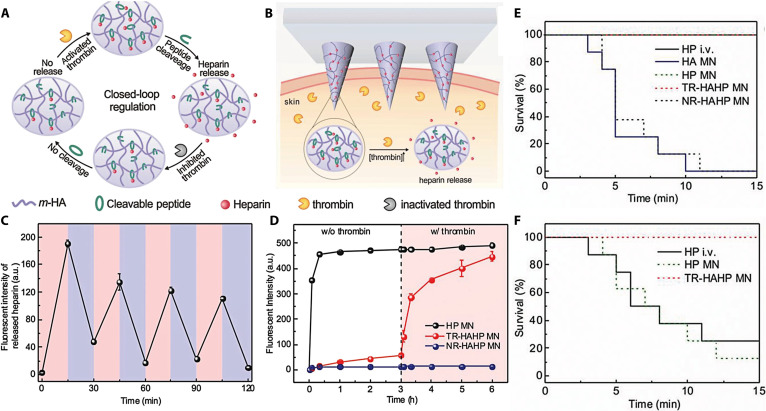
Schematic diagram and pharmacodynamic evaluation of a thrombin-responsive microneedle. (A) The operational principle governing the HP administration platform with self-regulating capability. (B) Illustration of the thrombin-sensitive TR-HAHP microneedle system. (C) Illustration of TR-HAHP microneedles upon thrombin exposure (light-colored profile: thrombin-absent condition; shaded profile: thrombin-present condition). (D) The controlled release of FITC-labeled HP from microneedles was investigated in thrombin solutions with varying concentrations. (E) Survival analysis of thrombin-treated mice. (F) Survival analysis of mice in a thrombosis induction model at 6 h after microneedle therapy. These figures were reproduced from Ref. [83] with permission.

Although HP can inhibit the formation of thrombus, excessive doses may impair normal coagulation function and cause spontaneous bleeding, whereas insufficient doses may fail to prevent the recurrence of thrombus. Therefore, Zhang et al. [83] developed a microneedle with thrombin-sensitive properties to achieve autonomous control of anticoagulation therapy (Fig. 7A and B). Briefly, hyaluronan was covalently linked to HP using a peptide substrate cleavable by thrombin, resulting in the formation of a thrombin-responsive needle matrix material (TR-HAHP). TR-HAHP was synthesized via polymerization under ultraviolet light irradiation. When thrombin is activated, the thrombolytic peptide can be cleaved, initiating the sustained liberation of HP from the TR-HAHP to exert long-term automatic regulation of coagulation and minimizing the risk of adverse spontaneous bleeding. In vitro drug release shows that TR-HAHP’s sensitivity to thrombin facilitated precise and reproducible HP delivery, with reduced release observed upon thrombin inhibition by initially liberated HP (Fig. 7C and D). The mice with acute thromboembolism could survive for 15 min after treatment with HP-loaded microneedles and TR-HAHP microneedles (Fig. 7E). Six hours after treatment, the survival rate of the mice treated with the TR-HAHP microneedle was significantly higher than that of the HP-loaded microneedle (Fig. 7F). These results indicate that TR-HAHP can timely release HP in response to elevated thrombin concentration, inhibiting coagulation over a long period.

Administration of conventional anticoagulant drugs like HP is prone to inducing adverse reactions such as thrombocytopenia and bleeding [84]. The development of bioactive substances that exhibit anticoagulant and antithrombotic properties similar to or even better than those of HP, yet with fewer side effects, is critical for the treatment of thrombosis [85]. Hirudin represents a recently identified antithrombotic polypeptide derived from the oral secretions of medicinal leeches. This bioactive compound comprises a chain of 65 to 66 amino acids and exhibits an approximate molecular mass of 7 kDa. Compared to HP, hirudin exerts stronger antithrombin activity that is not reliant on coagulation factors in plasma, avoiding possible platelet deficiency and lowering the likelihood of hemorrhage after administration. Recombinant bifunctional hirudin (RH) acts as a glycoprotein IIb/IIIa receptor antagonist that contains an Arg-Gly-Asp (RGD) sequence to further enhance anticoagulant activity [86]. Men et al. [87] developed recombinant hirudin-loaded insoluble microneedle patches (RHMNPs) to treat thrombotic diseases. In the murine model of acute pulmonary thromboembolism, the overall embolism degree of the high-dose drug-loaded microneedle cohort showed a statistically significant reduction compared to the untreated group. In addition, the anticoagulant activity observed in the microneedle-administered cohort showed comparable efficacy to conventional subcutaneous delivery, indicating that RHMNPs possess considerably prophylactic potential against sudden-onset pulmonary thromboembolism.

Fucoidan gum (FC) represents a class of sulfated carbohydrate polymers primarily derived from marine ecosystems [88] It is mainly composed of deoxyhexose residues, including fucose and additional simple sugars exhibiting diverse acetylation patterns and structural ramifications. FC exhibits comparable efficacy to HP in preventing blood coagulation and thrombus formation, while demonstrating fewer adverse reactions. It acts by blocking 2 key coagulation pathways: the tissue factor pathway and the contact activation pathway. However, FC is difficult to absorb but prone to degrade in the digestive tract, resulting in low oral bioavailability. In addition, intravenous administration of FC has a relatively high elimination rate, resulting in the rapid decrease in plasma concentration of FC. To address these dilemmas, Stephanie et al. [89] designed dissolving microneedles composed of gelatin and PVP to enable transdermal delivery of FC. Firstly, FC microneedles exhibited negligible hemolytic effects, confirming their biocompatibility for safe use. Moreover, the APTT in the FC microneedle-treated cohort was markedly prolonged compared to the HP-treated cohort, which was similar to the APTT of the intravenous injection group. These findings demonstrate that the percutaneous delivery of microneedle FC overcomes the constraints associated with oral and systemic FC delivery, effectively improving its antithrombotic efficacy.

## Application in the Treatment of Metabolic Diseases

Metabolic diseases encompass various chronic conditions arising from impairments in one or more facets of metabolism, the prevalence of which has steadily increased over the last 20 years, posing a substantial burden on global health [90]. In China, the prevalence of metabolic disorders stands at 31.1%, with an estimated affected population exceeding 450 million, and the associated risks of renal diseases, cardiovascular, and cerebrovascular diseases are progressively escalating [91]. Clinically prevalent metabolic diseases including diabetes, obesity, and osteoporosis, are all chronic in nature, often necessitating prolonged medication regimens [92]. Microneedles, as an easily manageable and configurable drug delivery platform, hold substantial market potential when integrated with biomacromolecules like peptides and polysaccharides, as delineated in Table 2.

**Table 2.** Recent studies on the microneedle-mediated delivery of biomacromolecules for metabolic diseases treatment

Metabolic disease	Types of biomacromolecules	Microneedle design	Main results	Reference
Diabetes	Protein	Coated microneedles made of stainless steel was developed to deliver proinsulin	Compared with conventional injection, coated microneedles activated stronger immune response, showing better therapeutic efficiency against type 1 diabetes	[148]
	Peptide	Glucose-responsive microneedle made of dopamine and 4-amino-3-fluorophenylboronic acid (AFBA) functionalized hyaluronic acid was developed to provide controlled release of insulin	The AFBA preferably binds to glucose in high glucose levels and increased hydrogel expansion and facilitating the secretion of insulin	[102]
Obesity	Peptide	A programmable microneedle comprising a PLGA shell with different molecular weights was developed to control the programmed release of somarlutide	This microneedle transformed subcutaneous injections of somarlutide per month into a single needle-free administration per month, effectively improving patient compliance	[117]
	Nucleic acid	Short hairpin RNA expression plasmid for FABP4 and FABP5 (sh (FABP4/5)) is assembled with arginine oligopeptides through electrostatic interaction to form SA-OPs, which was further loaded into the self-locking dissolving microneedles	The weight loss of obese mouse treated with SA-OPs-loaded microneedles showed a significant reduction without rebound in the 6 weeks after stopping treatment	[123]
Osteoporosis	Peptide	A separable microneedle composed of hyaluronic acid base and silk fibroin needles was designed for prolonged delivery of salmon calcitonin	The separable microneedle was more effective in promoting bone repair than subcutaneous injection	[129]

### Diabetes

Diabetes represents a chronic metabolic disorder marked by persistently elevated blood sugar levels. According to the global disease burden research statistics, the number of diabetes patients in the world reached 529 million in 2021, and it is projected that by 2050, the global diabetic population will exceed 1.31 billion [93]. The escalating trend in the incidence of diabetes has created a substantial demand for therapeutic drugs.

#### Insulin

Insulin, a 51-amino-acid protein, exerts its hypoglycemic effect by facilitating the uptake of glucose into hepatocytes, myocytes, and other tissue cells for the synthesis of glycogen, playing an important role in the management of diabetes [94]. Numerous glucose-sensitive materials designed for insulin administration have been developed to enable precise regulation of its release, with glucose binding protein (GBP) and glucose oxidase (GOx) being among the more commonly utilized [95]. GOx catalyzes the conversion of glucose into gluconic acid and hydrogen peroxide (H_2_O_2_), creating a local environment of hypoxia, acidity, and high H_2_O_2_, thereby stimulating the degradation of pH-responsive or hypoxia-responsive materials loaded with insulin and releasing insulin. In 2015, Yu et al. [96] developed the first hypoxia-responsive glucose-sensitive microneedle system by encapsulating insulin, GOx, and 2-nitroimidazole (NI)-conjugated hyaluronic acid (HS-HA) vesicles. Upon hyperglycemia, GOx-mediated glucose oxidation rapidly depletes local oxygen, generating a hypoxic microenvironment. This hypoxia triggers the enzymatic reduction of hydrophobic NI moieties on HS-HA to hydrophilic 2-aminoimidazoles, inducing rapid vesicle disassembly and subsequent insulin release. However, high concentrations of H_2_O_2_ often cause skin inflammation. To overcome this side effect, catalase is usually introduced into the system. For example, Chen et al. [97] designed a microneedle with a core–shell structure; it embeds catalase into the outer shell to eliminate H_2_O_2_ produced by GOx catalysis in the core and alleviate surrounding inflammation. Yang et al. [98] designed a glucose-responsive microneedle based on a multienzyme-metal organic framework (MOF), wherein GOx embedded within the MOF reacts with glucose under hyperglycemic conditions to generate gluconic acid and H_2_O_2_, creating an acidic milieu that prompts the disintegration of the MOF structure and the subsequent controlled release of insulin. Meanwhile, cobalt ions within the MOF mimic the function of catalase, decomposing excess H_2_O_2_ to prevent inflammation from occurring. Chen et al. [99] designed an insulin release mechanism modulated by blood sugar levels, utilizing the glucose transport protein (GLUT). They conjugated insulin with glucosamine and encapsulated it within erythrocyte-derived vesicles incorporating GLUT transporters and subsequently loaded it onto HA made microneedles. When interstitial fluid contains elevated levels of glucose, glucose competes with insulin for binding to GLUT, inducing a rapid release of insulin. In type 1 diabetic mouse models, the group treated with microneedles exhibited a swift reduction in blood glucose concentrations during the initial 60 min, maintained within the normal range for up to 5 h, demonstrating a more sustained effect compared to the subcutaneous injection of free insulin.

Both GOx-based and GBP-based controlled-release strategies necessitate intricate reactive components or vesicular encapsulation, which not only limit the loading capacity of insulin but also substantially augment the complexity of industrial production and clinical translation. Yu et al. [100] developed a coin-sized glucose-responsive microneedle (GR-MN) patch through in situ photopolymerization of a phenylboronic acid (PBA)-functionalized smart polymer. In hyperglycemic conditions, PBA-glucose binding generates negative charges, causing microneedle swelling and weakening electrostatic interactions with insulin to enable rapid release. Under normoglycemia, these reversible changes slow insulin release to prevent hypoglycemia. The 5-cm^2^ patch demonstrated rapid glucose-responsive insulin delivery in diabetic minipigs (>25 kg), maintaining normal blood glucose levels for over 20 h. Similarly, Zong et al. [101] developed a microneedle based on PBA-modified methacrylate HA. In low-glucose environments, PBA can tightly bind to gluconic acid-modified insulin (Gins), while in high-glucose environments, the affinity of PBA for glucose exceeds that for Gins, thereby triggering rapid insulin release. This design simplifies the fabrication process by eliminating the need for additional glucose-reactive components (e.g., nanoparticles or vesicles). Moreover, microneedles of ~1 cm^2^ can load 4 U of insulin and maintain normoglycemia in type 1 diabetic rat models for over 12 h. Liu et al. [102] designed a hydrogel microneedle with blood glucose-responsive swelling. The microneedle matrix is composed of an HA polymer functionalized with dopamine and 4-amino-3-fluorophenylboronic acid. Controllable insulin release in this system relies on the glucose concentration-dependent reversible crosslinking between dopamine’s catechol groups and boronic acid. When blood glucose levels decrease, the crosslinking weakens, slowing drug release to minimize hypoglycemia risks and enhance insulin safety. A glucose tolerance test was performed via intraperitoneal injection of glucose 2 h after hydrogel microneedle application or subcutaneous insulin injection. Diabetic rats treated with microneedles exhibited blood glucose normalization post-peak, comparable to healthy rats, whereas subcutaneously injected rats maintained hyperglycemia. These results confirm the hydrogel microneedles’ glucose-dependent drug release capability. In summary, PBA and its derivatives can also serve as exemplary glucose-responsive microneedle materials, facilitating straightforward and efficacious insulin therapy for diabetic patients.

To avert the severe hypoglycemia caused by frequent use of insulin or uncontrolled drug burst release, Yu [103] designed a microneedle for glucose-dependent glucagon release, utilizing the binding affinity between insulin and its corresponding aptamers. The underlying principle is that the aptamer–glucagon conjugate can bind with immobilized insulin on a methacrylate HA matrix, forming a conjugated HA substrate. Under conditions of high insulin levels, glucagon is promptly liberated from the bound HA network via displacement interactions involving both soluble and HA-fixed insulin molecules, thereby preventing hypoglycemia. After delivering an elevated insulin dose to type 1 diabetic mouse model, the glycemic concentration in the microneedle-treated animals gradually reverted to a hyperglycemic state, whereas the control group subjects succumbed to severe hypoglycemia.

Considering the degradation and solubility limitations of insulin in biodegradable polymers, Kim et al. [104] developed an implantable powder carrying microneedle (PCM) based on carboxymethyl cellulose (CMC). They used a solvent casting method to create a cavity in the center of the needle to load insulin powder. By adjusting the CMC concentration, the size of the cavity can be controlled, thereby controlling the loading amount of insulin powder. Under the same geometric structure, the drug encapsulation efficiency of PCM is approximately 150% higher than that of dissolving microneedles, indicating that PCM can break through the limitations of low solubility and activity loss, and significantly enhancing the insulin loading capacity at the microneedle scale at a lower preparation cost. After storage at 25 °C for 8 weeks, the insulin content in dissolving microneedles was 88.7% ± 3.0%, whereas that in PCM was 93.3% ± 3.8%, confirming that PCM has superior long-term stability compared to dissolving microneedles and potential for cold chain-free transportation and application.

Although traditional transdermal microneedle systems exhibit satisfactory performance in insulin-controlled release, their long-term application may lead to skin compliance issues. To address this limitation, Zhang et al. [105] developed an independent microneedle motor (IMNM) that enables efficient oral insulin delivery through a “magnetic navigation-tissue penetration-localized release” mechanism. The IMNM consists of a drug-loaded needle layer, a dissolvable intermediate layer, and a magnetically responsive base layer, encapsulated in enteric-coated capsules for gastric protection. Upon reaching the small intestine, the dissolved capsules release IMNM, which is then magnetically guided to the intestinal wall. The rapid dissolution of the intermediate layer facilitates the detachment of the needle layer and subsequent mucosal penetration, achieving deep insulin delivery. In diabetic rabbit models, magnetically guided IMNM demonstrated intestinal penetration exceeding 240 μm, significantly reducing blood glucose levels while maintaining prolonged glycemic control, which underscores its potential for oral biomacromolecular delivery.

#### GLP-1 receptor agonists

**Fig. 8.**
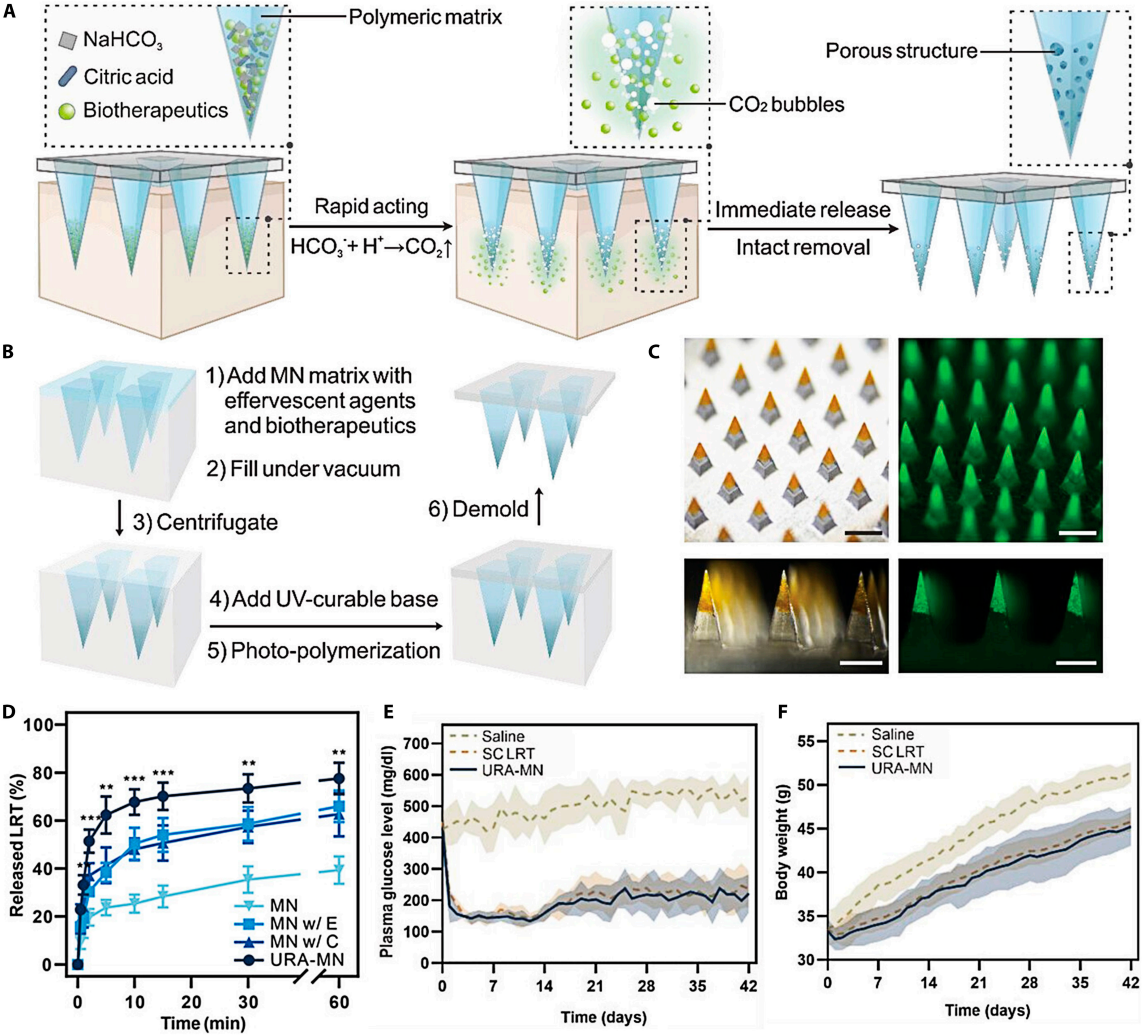
Composition, characterization, and pharmacodynamic evaluation of ultra-fast acting pneumatic microneedles. (A) The principle governing the exceptionally fast release of pharmaceuticals from microneedle. (B) Illustration depicting the manufacturing steps for the URA-MN. (C) Fluorescence microscopy images of the URA-MN. (D) Drug release curves of different microneedles (MN: microneedles without effervescent components or centrifugation; MN w/E: microneedles containing effervescent components; MN w/C: microneedles with centrifugation; URA-MN: microneedles with effervescent components and centrifugation). (E) Blood sugar concentrations in mice to varying interventions (Saline: saline group; SCLRT: subcutaneously delivered liraglutide group; URA-MN: pneumatic microneedles). (F) Weight change curves of mice in different treatment groups. These figures were reproduced from Ref. [107] with permission.

 Glucagon-like peptide-1 (GLP-1) receptor agonists are novel hypoglycemic drugs that have attracted much attention in recent years [106]. They lower blood glucose levels through multiple mechanisms, including promoting insulin release by pancreatic β cells, suppressing α cell production of glucagon, reducing food intake, and delaying digestive processes in the stomach. Unlike other hypoglycemic agents, the glucose-lowering action of GLP-1 agonists exhibits a blood sugar-dependent manner; GLP-1 receptor agonists alone do not cause hypoglycemia. You et al. [107] designed a pneumatic ultrarapid-acting microneedle (URA-MN) enabling rapid drug release for the delivery of liraglutide. When the effervescent agent in microneedles meets skin tissue fluid, it can produce gaseous bubbles, which accelerate therapeutic agent liberation and enhance percutaneous permeation, enabling complete administration within minutes and improving patient compliance (Fig. 8A to C). Pneumatic microneedles significantly accelerated drug release compared to non-pneumatic counterparts, achieving >60% release within 10 min (Fig. 8D). Animal experiments revealed a 33% inhibition of body mass increase and nearly 58% decline in glycemic levels after 6 weeks of continuous application (Fig. 8E and F). These results highlight the feasibility of pneumatic microneedles as an effective cutaneous administration system for biomacromolecules.

To solve the problem of frequent administration of exenatide, Chen et al. [108] designed a microneedle patch with hyperglycemic responsive drug release, which was loaded with mineralized GOx particles based on copper phosphate and mineralized exenatide particles based on calcium phosphate. The former acts as a “glucose sensor” to generate H^+^ through glucose oxidation reaction to lowering ambient pH, while the latter acts as a pH-responsive “drug depot” to degrade itself in a lower pH environment. Therefore, the microneedle patch can achieve intelligent and continuous drug release in the hyperglycemic environment, and its therapeutic effect is equivalent to that of long-acting exenatide preparation on the market, so that the blood sugar of diabetic mice can maintain normal levels within 5 to 6 days.

To minimize the loss of GLP-1 receptor agonist activity during preparation and storage and to achieve precise delivery into the skin, You et al. [109] designed an ovoid microneedle with a center of a liraglutide “yolk” layer and a yolk-covered eggshell to protect liraglutide from external pressure during preparation. Unlike traditional dissolved microneedles, ovate microneedles only require solely the penetration of the core yolk section into the dermis to achieve precise administration of liraglutide, and the depth and rate of microneedle insertion are not required. In type 2 diabetes murine models, both the microneedle-treated ova and subcutaneously administered cohorts were able to reduce hyperglycemia after 30 min of application and achieve similar blood glucose levels after 4 h, indicating that the ovum microneedle has a comparable drug delivery ability to the subcutaneous injection. In addition, liraglutide injection can only be stored at 4 °C for 30 days, while liraglutide supported by ovate microneedles can still maintain the same hypoglycemic ability as liraglutide injection after being stored at 4 °C for 2 months, confirming that ovate microneedles can maintain the stability of liraglutide during preparation and storage.

Oral administration is still the preferred route for individuals managing long-term conditions like diabetes, and it can lead to better treatment outcomes and patient compliance than percutaneous administration. To address the challenges associated with peptide and protein instability and poor intestinal permeability during oral delivery, Chen et al. [110] developed a dynamic omnidirectional mucosal adhesion microneedle system, inspired by the prickly head intestinal worm, for the delivery of semaglutide and its osmotic accelerator *N*-[8-(2-hydroxybenzoyl) amino] sodium caprylate. After delivering the tablets to the stomach, the system responds to environmental stimuli in the stomach before spraying the microneedle containing tablets into the stomach cavity, thereby reducing pre-exposure in the gastrointestinal tract. Microneedles on both sides of the tablet are used as dynamic omnidirectional adhesive systems (DOAMS) with a 2-layer design. The outer layer is Carbopol, a mucous membrane adhesive polymer, which has high bonding strength upon contact with wet tissue. The inner layer is thermoplastic polyester polycaprolactone, which can spontaneously swell and extend. In addition, they ensured that the microneedles have a hemispherical defect on one side, which unified the bending direction of the microneedles and gave the microneedles a higher tissue fixation ability, so that the sprayed tablets can adhere to the stomach mucosa instantaneously. In porcine studies, wash tests revealed that while conventional tablets migrated > 30 mm within 20 s, the DOAMS modified tablets remained firmly in place. Furthermore, the absorption rate of DOAMS tablets in pigs was significantly higher, and the peak blood concentration was twice that of the traditional tablets.

Cutaneous wounds represent the predominant factor leading to limb loss in diabetic patients, resulting in severe psychological and physical trauma to people with diabetes [111]. Typically, diabetic cutaneous lesions arise from persistent wound healing impairments in diabetes, presenting with local hyperglycemia, chronic inflammation, and repeated infections [112]. Zhang et al. [113] prepared an autonomous enzyme-integrated microneedle for the rapid healing of diabetic wounds. The system consists of anodic microneedles containing GOx and cathodic microneedles containing horseradish peroxidase, which can trigger the enzyme-linked reaction through the local hyperglycemic environment of the wound, alleviate elevated glucose levels in the injured area while producing a consistent and prolonged electrical stimulation to accelerate wound healing, and prevent scar formation. In the rat model of type 1 diabetes, wounds of rats in the enzyme-linked microneedles group healed completely (0%) after a 21-day intervention, whereas the untreated group showed 16.6% unhealed lesion. In addition, immunoassay analysis showed that the enzyme-linked microneedles group had the lowest levels of inflammatory factors, indicating that it also had a significant inhibitory effect on inflammatory response.

### Obesity

Obesity poses a substantial health burden in contemporary society. Worldwide obesity rates have increased nearly 3-fold over the past 5 decades [114]. Global statistics from WHO indicate that approximately 13% of the adult population suffer from obesity, and it is recognized as a key determinant of fatal outcomes and reduced quality of life. As a chronic metabolic disease, obesity markedly elevates the likelihood of developing malignancies, diabetic conditions, and heart-related ailments. It is estimated that 4% to 9% of patients diagnosed with cancer are ascribed to excess body fat, and the life expectancy of obese people is shortened by 5 to 20 years [115]. Traditional obesity treatments include dietary control, physical exercise, and surgery. Although these approaches are effective, they rely heavily on individual self-discipline and can cause serious side effects [116].

**Fig. 9.**
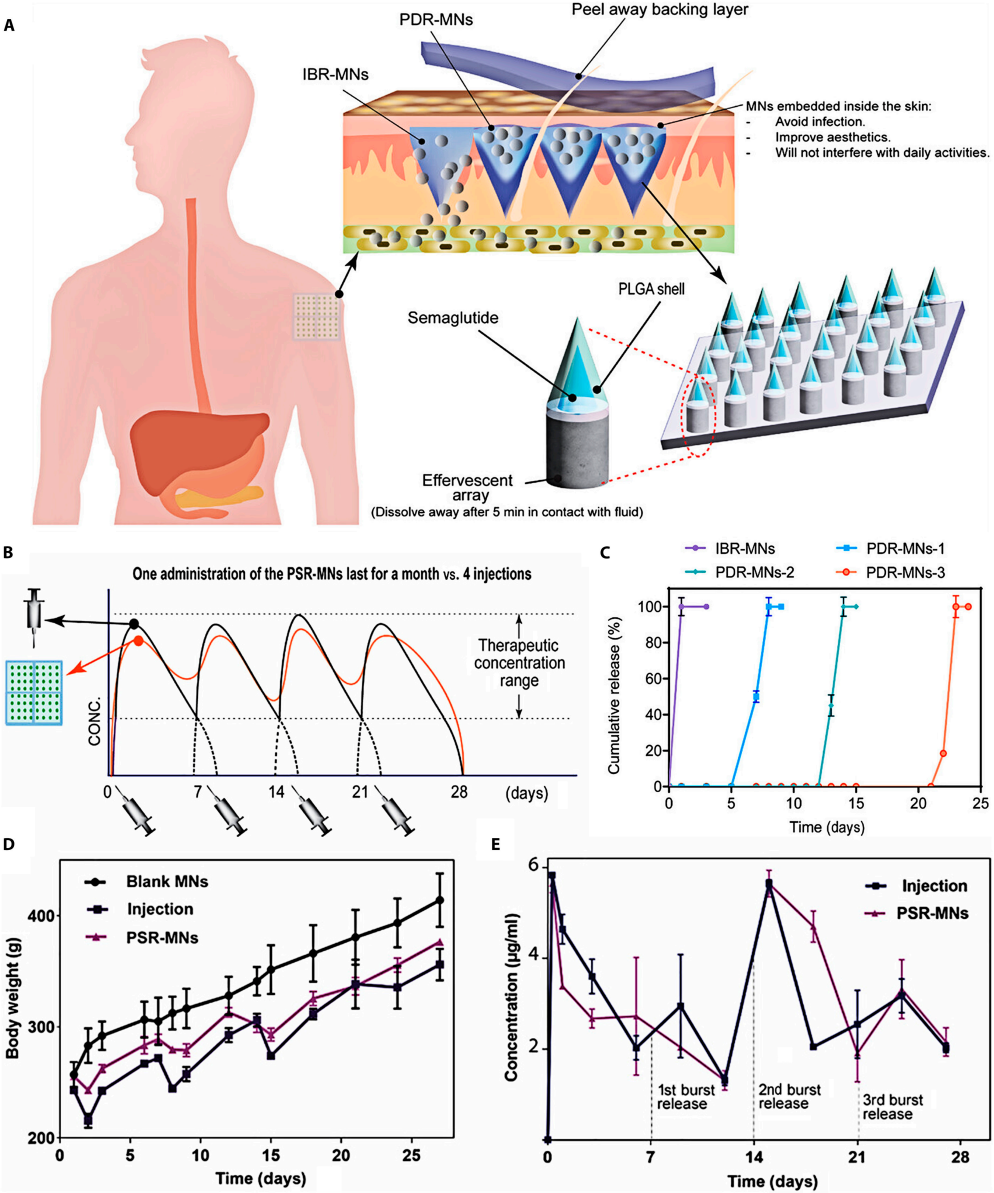
Schematic diagram and pharmacodynamic evaluation of a programmable microneedle. (A) Mechanism of a programmable microneedle. (B) Schematic diagram of programmed release of microneedles. (C) In vitro release kinetics of a programmable microneedle. (D) Weight change curves of obese rats of different groups. (E) Plasma drug concentration versus time curves of subcutaneous injection and programmable microneedle. These figures were reproduced from Ref. [117] with permission.

Remarkable advancements have been achieved in the formulation of weight-loss medications, with GLP-1 receptor agonists standing out [116]. Typically, the biological half-life of GLP-1 receptor agonists ranges between 7 and 13 h, and they require daily subcutaneous injections for multiple months, resulting in low patient compliance and high economic cost. Therefore, scientists have sought to develop various microneedles as an alternative delivery approach for administering GLP-1 receptor agonists in obesity management. Singh et al. [117] designed a programmable microneedle (PSR-MN) that can maintain the plasma concentration of semaglutide required for treatment for 1 month through a single dose (Fig. 9A). The PSR-MN system comprises 4 1-cm^2^ patches, one of which releases semaglutide initially, and the others are programmed to release semaglutide loaded in the core at 7, 14, and 21 days post-administration, respectively, by adjusting the molecular weights of PLGA to control the degradation behavior of the needle shell (Fig. 9B and C). In addition, an effervescent baseplate consisting of PVP, citric acid, and sodium bicarbonate was designed to achieve rapid needle–baseplate separation, as the effervescent agents underwent chemical reaction to generate gas bubbles upon contact with skin interstitial fluids. In vivo pharmacokinetic studies revealed that the plasma concentration of semaglutide following a single administration of PSR-MN was comparable to that of multiple subcutaneous injection. It indicated that PSR-MN transforming 4-round subcutaneous injection of semaglutide per month into a single needle-free administration per month can well improve the compliance of patients with obesity (Fig. 9D and E).

Juhng et al. [118] developed a triple-layer microneedle (TLM) system for long-term delivery of liraglutide. The TLM consists of a base layer, a core layer, and a shield layer, with all drugs encapsulated in the core layer and protected by the base and shield layers, which not only effectively prevent drug deformation or degradation but also ensure that only the core layer is inserted subcutaneously for full drug delivery without requiring high insertion depth. After daily topical application of TLM for 2 weeks, the body mass of the mice receiving hypodermic administration and microneedle decreased by 20%. In addition, the gonadal adipose tissue exhibited markedly less lipid deposition in both subcutaneous and microneedle-treated groups. Collectively, these results demonstrate that the liraglutide-loaded TLM exhibits promising therapeutic prospects for managing obese individuals.

**Fig. 10.**
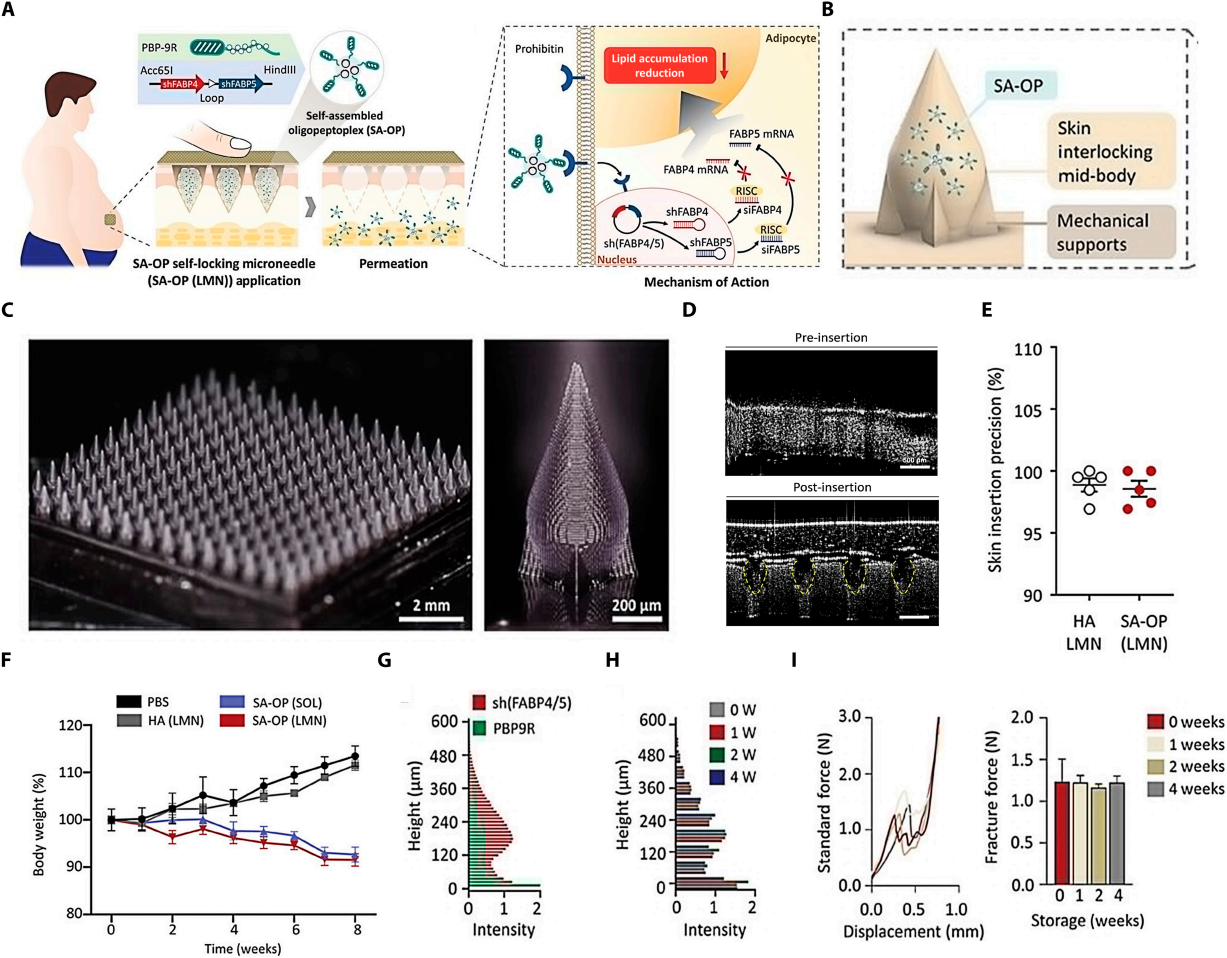
Composition, characterization, and pharmacodynamic evaluation of SA-OP (LMN)s. (A) Therapeutic mechanism of SA-OP (LMN)s. (B) Composition of self-locking dissolving microneedles. (C) Geometry of a self-locking dissolving microneedle. (D) OCT cross-sectional images of SA-OP (LMN)s. (E) Accuracy evaluation of HA LMNs and SA-OP (LMN)s implantation in porcine cadaver skin. (F) Weekly body weight measurements [PBS: PBS-treated control group; HA LMN: SA-OP-unloaded LMN group; SA-OP (SOL): subcutaneously injected SA-OP (SOL) group; SA-OP (LMN)s: SA-OP-loaded LMN group]. (G) The luminescence levels of nucleic acids and protein fragments in SA-OP (LMN) samples following a 4-week preservation period. (H) Assessment of fluorescence signal variations in gene–peptide over a 28-day preservation period. (I) Evaluation of typical strength (left) and break resistance (right) for SA-OP (LMN) samples across a 28-day preservation interval. These figures were reproduced from Ref. [123] with permission.

High doses of GLP-1 can activate GLP-1 receptors in the brain, thereby suppressing appetite, inducing satiety, and diminishing fat absorption. However, long-term use of GLP-1 agonists may induce neurological diseases and steatorrhea and is prone to cause weight relapse after drug withdrawal [119]. Previous research has demonstrated that genetic interventions specifically targeting adipose tissue can effectively modulate the metabolic processing of fats [120], such as inhibiting fatty acid binding protein (FABP) expression in fat cells by RNA interference, thereby blocking lipid accumulation [121,122]. For instance, Choi et al. [123] constructed a plasmid vector expressing 2 distinct shRNAs targeting both FABP4 and FABP5 [sh (FABP4/5)], which was self-assembled into nano-oligopeptides (SA-OPs) through electrostatic interaction with positively charged arginine oligopeptides (PBP9R). The SA-OPs were then loaded in the self-locking microneedles by 3D printing technology. The self-locking microneedles feature a distinctive tapered design, with a sharp 2-μm apex gradually widening to a 340-μm central section before narrowing to a 250-μm proximal end (Fig. 10A to C), which leads to a highly stable and accurate rate of cutaneous permeation and attachment (Fig. 10D and E). Relative to the control group without intervention, administration of SA-OP-laden microneedle experienced significant weight loss after 6 weeks of treatment without a reduction in food intake (Fig. 10F). To avoid the clustering of genes with PBP9R, nucleic acids and short-chain peptides should be maintained in isolated freeze-dried preparations and combined solely at the time of administration. Notably, this microneedle system significantly enhanced their long-term stability and maintained their particle size at 4 °C for up to 4 weeks (Fig. 10G to I).

### Osteoporosis

Osteoporosis is a systemic metabolic disease characterized by decreased bone mass and deterioration of bone microstructure. Abnormal renal metabolism or calcium and phosphorus metabolism, which is one of the most common metabolic disorders in orthopedics, can lead to skeletal system diseases and substantially increase the risk of bone fragility and fracture. Osteoporosis currently affects over 200 million individuals worldwide, with an annual incidence of 8.9 million osteoporosis-associated pathological fractures—equivalent to one fracture occurring every 3 s [124–126].

#### Salmon calcitonin

Salmon calcitonin is a polypeptide chain composed of 32 amino acids that can inhibit osteoclast activity and is clinically administered via injection or nasal spray to treat osteoporosis. However, frequent injection causes allergy and side effects, and nasal administration suffers from poor bioavailability and patient compliance [127]. To solve these problems, researchers have developed several microneedles to deliver salmon calcitonin for osteoporosis treatment. For example, Tas et al. [128] developed coated microneedles to deliver salmon calcitonin. The drug delivery efficiency of the encapsulated microneedle showed no significant difference from that achieved via subcutaneous injection, yet it demonstrated a 13-fold increase compared to intranasal delivery. To achieve prolonged therapeutic effect, Li et al. [129] fabricated a detachable microneedle system utilizing silk fibroin for the needle components and HA for the supporting matrix, designed to achieve prolonged delivery of salmon calcitonin. Pharmacodynamic studies further showed that the sustained microneedle was more effective in promoting bone repair than subcutaneous injection. Compared with unmodified silk fibroin, the modified microneedle enabled the cumulative drug release to be decreased by 24.53%.

#### Human parathyroid hormone

**Fig. 11.**
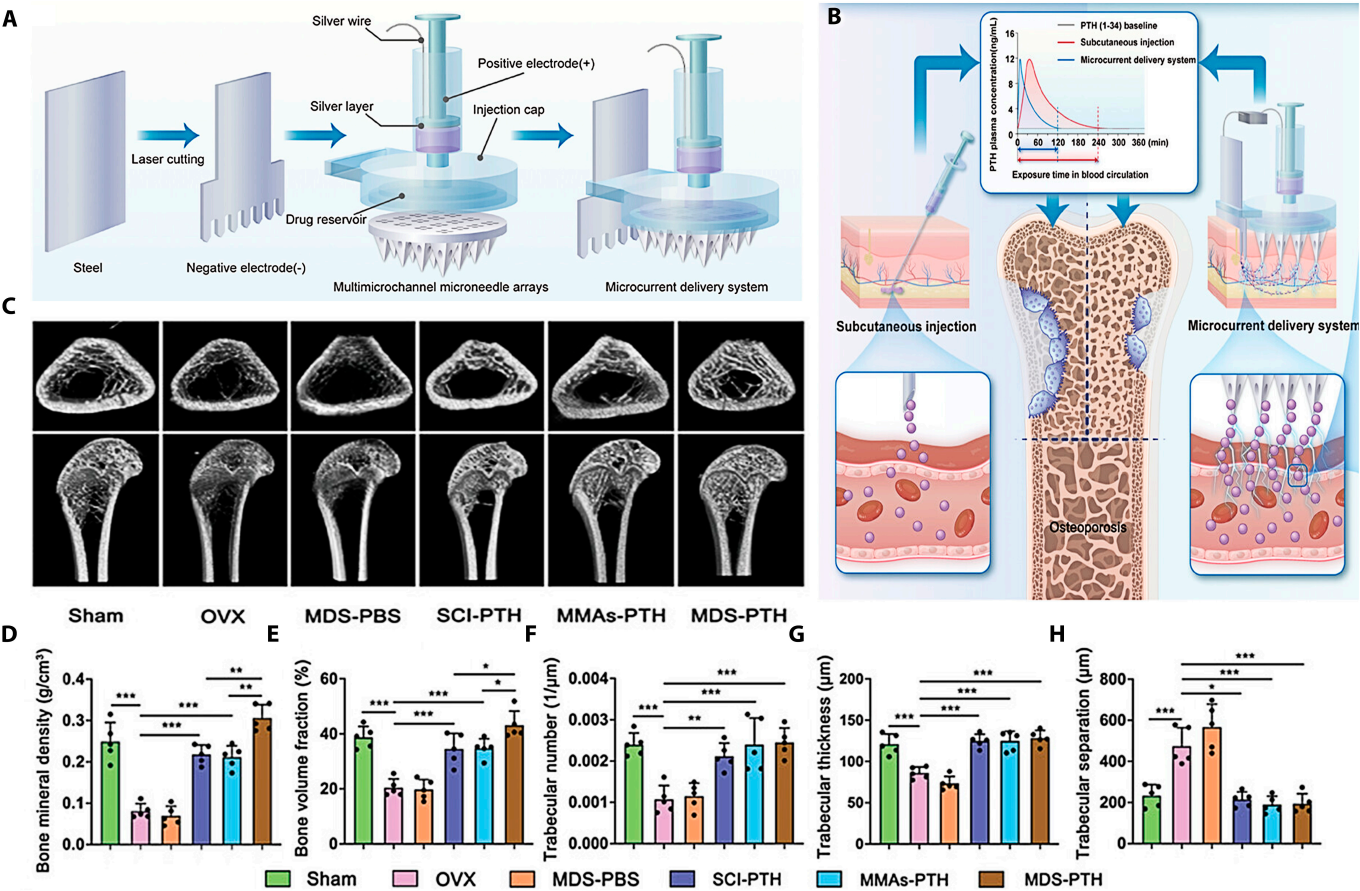
Composition, characterization, and pharmacodynamic evaluation of multi-microchannel microneedle array. (A) Components of the integrated microcurrent delivery system. (B) Schematic diagram of the microcurrent delivery system. (C) 3D modeling of femoral condyles across various cohorts (Sham: Sham operation group; OVX: osteoporosis model group; MDS-PBS: MDS loaded with PBS; MMAs-PTH: MN loaded with PTH without microcurrent; MDS-PTH: MDS loaded with PTH with microcurrent. (D) Quantitative results of bone mass density, (E) volumetric bone proportion, (F) count of trabeculae, (G) thickness of trabecular structures, and (H) spacing between trabeculae. These figures were reproduced from Ref. [130] with permission.

Human parathyroid hormone (1–34) (PTH) manifests osteoanabolic and osteocatabolic properties, wherein shorter intervals of plasma exposure tend to promote bone formation. PTH is clinically administered by subcutaneous injection and has the following drawbacks: limited passive diffusion, hindered absorption by the vascular endothelial barrier, and low delivery efficiency. These limitations often cause long drug exposure time and reduced bone anabolic effect. To enhance drug delivery efficiency and safety of administration, Mo et al. [130] developed a microcurrent delivery system (MDS) to deliver PTH. MDS is composed of a multi-microchannel microneedle array (MMA), a syringe positive electrode, a counter electrode microneedle as the negative, and a protective cap for injection (Fig. 11A). When the MMA penetrates skin and contacts physiological fluid, an electrical pathway is formed connecting the anode, medicinal liquid, tissue fluid beneath the skin, and cathode. Then, a microcurrent is generated to enhance the migration of cationic PTH molecules, increase vascular endothelial permeability, promote the uptake of PTH, and reduce its duration of contact (Fig. 11B). In the osteoporosis rat model, compared with subcutaneous injection, the PTH delivered by MDS more efficiently inhibited osteoclast differentiation, significantly enhancing the bone anabolic effect (Fig. 11C to H).

#### Teriparatide acetate

Teriparatide acetate (TA), a synthetic analog of human parathyroid hormone 1–34, functions as a bone-building medication through stimulating osteoblast activity, thereby enhancing skeletal growth. It is employed as an innovative treatment option for osteoporosis. Currently available TA products require patients to receive subcutaneous injections once a week or once a day, showing poor patient compliance. Sim et al. [131] designed a dissolvable microneedle array delivering weekly administered teriparatide for enhanced osteoporosis therapy. After a 5-min exposure to the TA-DMN patch, approximately 87.6% of the TA was successfully transported across the porcine skin barrier. The microneedle-delivered TA exhibited 66.9% bioavailability, with efficacy comparable to subcutaneous injections. The findings demonstrate that microneedles loaded with TA are promising for treating osteoporosis without causing any patient discomfort or safety issues.

## Application in the Treatment of Autoimmune Diseases

Autoimmune diseases arise from a breakdown in the immune system’s ability to distinguish self from non-self, resulting in harmful immune reactions against the body’s own tissues. These conditions are associated with considerable morbidity and mortality [132]. Approximately 5% to 9% of the global population is impacted by autoimmune disorders globally, including as high as 5% to 8% in the United States, and the affected population approaches 40 million in China [133]. At present, there are more than 100 known autoimmune diseases, and the common autoimmune diseases in the clinic include rheumatoid arthritis (RA), multiple sclerosis, and psoriasis, among others. Most of these diseases have unclear etiology, are prone to relapse, require long-term care and treatment, and cause a huge medical burden on the lives of patients [134]. The common treatment methods for autoimmune diseases include general supportive care and pharmacological therapy. Among them, the biomacromolecular-specific immunosuppressants such as monoclonal antibodies (mAbs) and bioactive peptides have higher safety, specificity, and effectiveness than nonspecific immunosuppressants such as tacrolimus and azathioprine, and have great prospects in the treatment of autoimmune diseases [135]. To enhance the therapeutic effect, improve the standard of living of patients, and reduce the medical burden, increasing research attention has been directed toward microneedles for the administration of immunosuppressive agents, as shown in Table 3.

**Table 3.** Recent studies on the microneedle-mediated delivery of biomacromolecules for the treatment of autoimmune disease

Autoimmune disease	Types of biomacromolecules	Formulation design	Main features	Reference
Rheumatoid arthritis	Protein	Dissolving microneedles loaded with TNF-α inhibitor etanercept was developed to treat rheumatoid arthritis with improved patient compliance	Etanercept delivered by microneedle produced comparable bioavailability to subcutaneous injection	[140]
	Peptide	Photo-crosslinked microneedles made of MeHA were developed to provide sustained release of melittin and enhance drug delivery efficiency	Compared to HA microneedles, MeHA microneedles prolonged the release and retention of melittin in the skin to exert better therapeutic efficacy against inflammation	[139]
Psoriasis	Peptide	Photothermal dissolving microneedles co-loaded with MXene and IL-17 mAb was developed for the treatment of psoriasis	The dissolution of microneedles was accelerated to trigger the release and permeation of IL-17 mAbs upon NIR, thereby increasing the quantity of drug reaching the affected skin areas in psoriasis	[143]
	Protein	Highly swellable hydrogel microneedles were developed to co-deliver methotrexate and IL-17 for the management of psoriasis	The hyperinflated microneedles served as an implantable drug reservoir to continuously release drugs, significantly improving drug delivery efficacy	[142]
	Protein	Dissolving microneedles co-loaded with NLRP3 targeting Cas9 and dexamethasone nanoparticles were developed for the management of psoriasis	The microneedles integrating gene and pharmaceutical agents significantly repaired skin barrier and reduced skin inflammation levels	[144]

### Rheumatoid arthritis

RA is a prevalent chronic autoimmune disorder characterized by proliferative inflammation in the synovium, cartilage erosion, and joint damage. Worldwide, RA affects approximately 1 in 200 to 1 in 100 individuals, and there is a huge demand for clinical treatment [136]. Traditional therapeutic regimens for RA are usually performed by oral or injectable administration of chemicals including nonsteroidal anti-inflammatory medications and corticosteroids. Although these chemicals may provide short-term symptom alleviation by suppressing immune responses and reducing inflammation, they fail to halt disease progression or protect joints from deterioration [137]. In recent years, biomacromolecule-based immunotherapy like tumor necrosis factor-α (TNF-α) inhibitors and melittin, which targets specific pathogenic pathways and restores immune homeostasis, has shown promising clinical potential in RA [138].

To improve patient compliance, researchers have attempted to use microneedles to deliver biologic agents for the immunotherapy of RA. In view of the autoimmune regulation disorder of RA, Du et al. [139] synthesized methacrylate-modified HA to prepare photo-crosslinked microneedles with prolonged drug release, enabling cutaneous administration of melittin. Cyanine 5-labeled melittin was loaded into the microneedles to track its cutaneous retention following delivery via microneedles. Relative to the model and subcutaneous injection groups, the microneedles loaded with melittin demonstrated sustained cutaneous drug persistence for 7 days, while free drugs were cleared within 3 days. Moreover, these microneedles markedly reduced the concentrations of interleukin-17 (IL-17) and TNF-α in both joints and serum. Cao et al. [140] designed microneedles composed of hyaluronan to administer the TNF-α inhibitor etanercept. Relative to the subcutaneous injection group, the microneedle group had comparable bioavailability and a higher level of patient compliance.

### Psoriasis

Psoriasis represents a persistent inflammatory disorder of the skin, primarily manifesting as scales, erythema, and papules. Psoriasis is affecting approximately 125 million individuals globally, which may cause itching of the skin, and lead to a series of complications including metabolic abnormalities and cardiac and cerebral vascular conditions, profoundly impairing both physical and mental well-being in affected individuals [141].

**Fig. 12.**
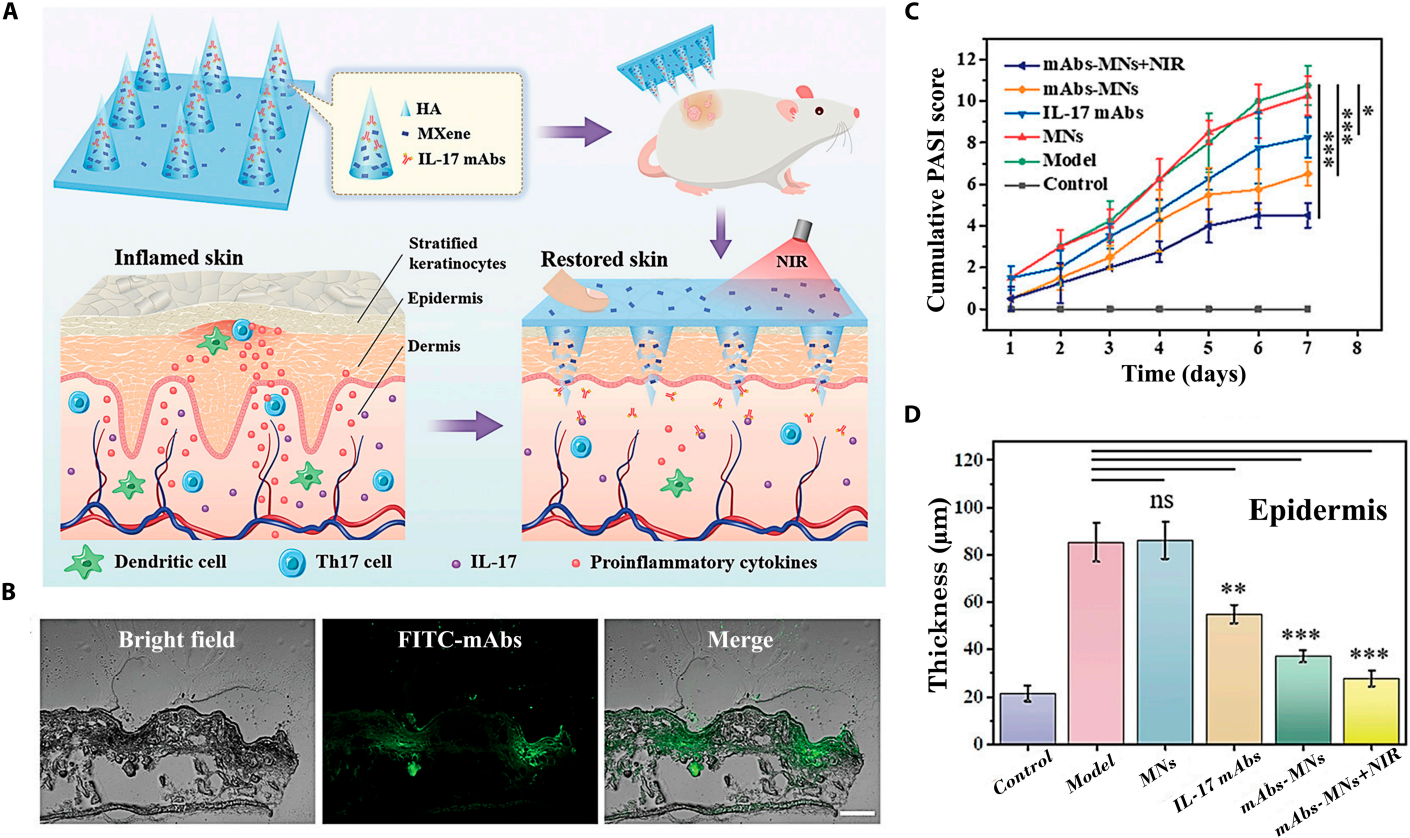
Composition, characterization, and pharmacodynamic evaluation of a photothermal responsive microneedle. (A) Schematic illustrating the therapeutic mechanism of IL-17 mAbs-encapsulated photothermally activatable microneedle for managing psoriatic lesions. (B) Fluorescence microscopy visualization of IL-17 mAbs conjugated with FITC released from a microneedle upon NIR irradiation. (C) The PASI scores of mice in various intervention cohorts. (D) Dermal layer measurements across different mouse cohorts (Model: imiquimod treatment; MNs: blank MN; mAbs-MNs: MN encapsulated IL-17 mAbs; mAbs-MNs+NIR: MN encapsulated IL-17 mAbs upon NIR exposure). These figures were reproduced from Ref. [143] with permission.

Microneedle-mediated topical delivery of biomolecules that intervenes the inflammatory signaling pathway has brought new development for the treatment of psoriasis, breaking the dilemma of traditional treatments such as difficult absorption and side effects. Li et al. [142] developed hydrogel microneedles exhibiting a high swelling rate, which were fabricated for the delivery of methotrexate and IL-17 in the treatment of psoriasis. The delivery of IL-17 via this microneedle device was 1.5 to 2 times more effective than subcutaneous injection at the same dose of IL-17. Moreover, microneedle delivery of methotrexate was 2 to 3 times more effective in reducing the number of mast cells and epidermal thickness compared with conventional oral or applied methotrexate at the same dose. In another study, Wu et al. [143] designed photothermal dissolving microneedles to deliver photothermal agent MXene and IL-17 mAb for the treatment of psoriasis. When exposed to NIR light, MXene converts light into heat to cause a surge in local temperature, allowing the microneedles to quickly dissolve and deliver IL-17 to the skin (Fig. 12A and B). Compared to subcutaneous injection, IL-17-loaded microneedles provide comparable efficacy at reduced doses and help reduce side effects (Fig. 12C and D), showing great potential for the treatment of psoriasis.

Clustered regularly interspaced short palindromic repeats (CRISPR) is a commonly used genome modification tool in the biomedical field and has been widely used in a variety of diseases. Wan et al. [144] developed dissolving microneedles co-loaded with CRISPR-associated protein 9 (Cas9) and dexamethasone nanoparticles for the management of psoriasis. Cas9 is capable of specifically modulating the NLRP3 inflammasome, a multiprotein complex linked to NOD-like receptor pathways, thereby mitigating inflammatory responses. Compared to dexamethasone cream and tacrolimus ointment, microneedle-mediated treatment demonstrated superior enhancement of the epidermal barrier properties, maintained the integrity of the skin structure, and reduced the levels of proinflammatory cytokines such as IL-1β, IL-18, and IL-17. The combination of Cas9 and microneedles may provide new ideas for the treatment of other inflammatory skin diseases.

## Conclusion and Prospect

In recent years, scientists have continuously strived to advance the medical application of microneedles. As shown in Table 4, more than 30 microneedle products have progressed to advanced clinical testing stages (Phase II/III) globally, including those incorporating biomacromolecules, such as insulin, abaloparatide, and monoclonal antibody, which make up the majority of these products. These evidences demonstrate the great potential of microneedles in painless delivery of biomacromolecules for vaccination, percutaneous immunotherapy, and gene therapy, opening up a new avenue for major disease treatment.

**Table 4.** Currently active clinical trials of microneedle-mediated drug delivery systems

Type of diseases	Medicinal substance	Categories of microneedles	Microneedles material	CT staged	NCT identifier
Post-surgical scars	siSPARC	Dissolving microneedles	–	2	NCT06138964
Influenza	Trivalent influenza vaccine	Hollow microneedles	–	1/2	NCT01707602
Influenza	Intanza	Microneedle injection system	–	4	NCT01368796
Renal failure	Hepatitis B vaccination	Novel intradermal microneedles	Silicone	2/3	NCT02621112
COVID-19	Comirnaty vaccine (Pfizer)	Nanoporous microneedles	–	2	NCT05315362
Varicella Zoster infection	Zostava	Novel intradermal microneedle	–	2/3	NCT02329457
Cutaneous squamous cell cancer	Doxorubicin	Dissolving microneedles	–	1/2	NCT05377905
Nodular basal cell carcinoma	Doxorubicin	Dissolving microneedles	–	2	NCT06608238
Basal cell cancer	Doxorubicin	Dissolving microneedles	–	1/2	NCT04928222
Ovarian carcinoma	Modi-1/Modi-1v vaccines	MicronJet600 (hollow microneedles)	–	1/2	NCT05329532
Uveitis	Triamcinolone acetonide	Microinjector	–	1/2	NCT01789320
Diabetes	Insulin	Hollow microneedles	Borosilicate glass pipettes	2/3	NCT00837512
Diabetes	Insulin and glucagon	MicronJet (hollow microneedles)	Silicone	1/2	NCT01684956
Diabetes	Insulin	Hollow microneedles	–	2	NCT01061216
Measles and rubella	Measles/Rubella vaccine	Dissolving microneedles	–	1/2	NCT04394689
Migraine	Zolmitriptan	MicronJet600 (hollow microneedles)	Titanium	2/3	NCT02745392
Auto-immune/auto-inflammatory diseases	Adalimumab	MicronJet600 (hollow microneedles)	Silicone	1/2	NCT03607903
Osteoporosis	Abaloparatide	Coated transdermal microarray	–	2	NCT01674621
Postmenopausal osteoporosis	Abaloparatide	Solid microstructured transdermal system	–	3	NCT04064411
Gingival recession	Platelets rich in fibrin and vitamin C	DermaPen Ultima M8	Titanium	4	NCT05841303
Esophageal cancer	Tinidazole	Dissolving microneedles	–	3	NCT06418945
Topical analgesia	Lignocaine	Dissolving microneedles	–	1/2	NCT05694858
Topical analgesia	Lidocaine	Coated microneedles	–	2/3	NCT01145326
Poliomyelitis	Fractional IPV	MicronJet600 (hollow microneedles)	Silicone	3	NCT01813604
Actinic keratosis	ALA	Dissolving microneedles	–	2	NCT02632110
Enlarged facial pores	Botulinum toxin	DermaPen Ultima A6	Titanium	3	NCT06293755
Migraine	Zolmitriptan	Qtrypta	Titanium	3	NCT03282227
Antibiosis	Metronidazole	Solid microneedles	–	4	NCT05929794
HIV-infected subjects	Inactivated polio vaccine	MicronJet600 (hollow microneedles)	Silicone	2	NCT01686503
Crow’s feet wrinkles	TEOSYAL	MicronJet (hollow microneedles)	Silicone	4	NCT02497846
Skin inflammation	Dupilumab	Coated microneedles	Poly-l-lactide	2/3	NCT05535738

The common goal of scientific research institutions and pharmaceutical enterprises is to promote the microneedle products from bench to bedside. However, commercial microneedle products remain absent from current markets and the practical application and clinical translation of microneedles is still facing several major challenges. First, the drug dosage delivered via microneedles may be imprecise, owing to the difference in skin structure and administration mode of the individual. Besides, the drug loading capacity of microneedles is quite low, making it challenging for a single administration to meet the clinical dosage requirements. Further, the safety issues caused by long-term use of microneedles such as granuloma formation and pigment deposition have not been well addressed yet, limiting the practical application of microneedles. To address these challenges, researchers are refining the design and manufacture of microneedles through interdisciplinary collaboration and introduction of advanced technologies. For instance, the high viscosity of the needle matrix solution is the key factor leading to the reduced drug encapsulation efficiency within microneedles, because it makes the microperfusion of needle solution difficult to fill in the mold. The drug loading capacity of microneedles can be efficiently increased by optimizing the geometric parameters of microneedles (such as microneedle shape, needle tip density, and needle length), formulation compositions (such as the concentration or ratio of drug and polymer), preparation process (laser, micro-electro-mechanical systems processing, and chemical etching), and preparation method (centrifugal micro-perfusion, vacuum micro-perfusion, and 3D printing [145]).

The industrial production of microneedles is a prerequisite for the commercialization of microneedle products, but it still faces many challenges, especially for the manufacture of biomacromolecule-laden microneedles: (a) biomacromolecules have inferior stability to small molecules, posing a challenge in preserving their bioactivity throughout manufacturing; (b) the standardized manufacturing process of microneedles lacks distinct regulations by GMP, making it difficult for one to obtain approval from the drug regulatory agency; and (c) the quality standards of microneedles still lack corresponding policies and regulations for reference. Nanotechnology encapsulation or novel mild manufacture methods have been suggested to enhance the structural integrity of biomacromolecules. In addition, scientists focus more on balancing stability, efficacy, and safety in microneedle design. Furthermore, collaboration from pharmaceutical companies, government agencies, and academic research institutions is still required for establishing policies and overcoming bottlenecks in industrial translation.

The integration with artificial intelligence, functional biomaterials, and advanced biosensing technologies propels the swift progression of microneedles. In the last 20 years, the scientific literature and patents on microneedles have grown exponentially. Researchers are boldly leveraging their abundant imagination to investigate the potential use of microneedles across various challenging medical conditions, such as developing microneedle tacks, microneedle robots, and biomimetic microneedles [146,147]. The inspiring achievements obtained from extensive and cross-cutting collaborative researches have greatly promoted the onset of microneedle technology and its therapeutic utilization for major diseases.

## References

[1] CheX G, Kang W, Li W, Chen S, Gao Y. Oral delivery of protein and peptide drugs: From non-specific formulation approaches to intestinal cell targeting strategies. Theranostics. 2022;12(3):1419. 35154498 10.7150/thno.61747 PMC8771547

[2] FDA. Novel drug approvals for 2024. 2024.

[3] Mahato RI, Narang AS, Thoma L, Miller DD. Emerging trends in oral delivery of peptide and protein drugs. Crit Rev Ther Drug Carrier Syst. 2003;20(2–3):153–214. 14584523 10.1615/critrevtherdrugcarriersyst.v20.i23.30

[4] Cao S, Xu S, Wang H, Ling Y, Dong J, Xia R, Sun X. Nanoparticles: Oral delivery for protein and peptide drugs. AAPS PharmSciTech. 2019;20(5):190. 31111296 10.1208/s12249-019-1325-z PMC6527526

[5] Bedir T, Kadian S, Shukla S, Gunduz O, Narayan R. Additive manufacturing of microneedles for sensing and drug delivery. Expert Opin Drug Deliv. 2024;21(7):1053–1068. 39049741 10.1080/17425247.2024.2384696

[6] Panda A, Matadh VA, Suresh S, Shivakumar HN, Murthy SN. Non-dermal applications of microneedle drug delivery systems. Drug Deliv Transl Res. 2022;12(1):67–78. 33629222 10.1007/s13346-021-00922-9

[7] Zhou R, Yu J, Gu Z, Zhang Y. Microneedle-mediated therapy for cardiovascular diseases. Drug Deliv Transl Res. 2022;12(2):472–483. 34637115 10.1007/s13346-021-01073-7

[8] Rzhevskiy AS, Singh TRR, Donnelly RF, Anissimov YG. Microneedles as the technique of drug delivery enhancement in diverse organs and tissues. J Control Release. 2018;270:184–202. 29203415 10.1016/j.jconrel.2017.11.048

[9] Ferreira LEN, Franz-Montan M, Benso B, Gill HS. Microneedles for oral mucosal delivery—Current trends and perspective on future directions. Expert Opin Drug Deliv. 2023;20(9):1251–1265. 37781735 10.1080/17425247.2023.2264189

[10] Zhang X, Li M, Gao Q, Kang X, Sun J, Huang Y, Xu H, Xu J, Shu S, Zhuang J, et al. Cutting-edge microneedle innovations: Transforming the landscape of cardiovascular and metabolic disease management. IScience. 2024;27(9):110615. 39224520 10.1016/j.isci.2024.110615 PMC11366906

[11] Han J, Wu Z, Zhan S, Sheng T, You J, Yu J, Fu J, Zhang Y, Gu Z. Biorhythm-mimicking growth hormone patch. Nat Mater. 2025;24(8):1283–1294. 40181125 10.1038/s41563-025-02188-9

[12] Advances in Transdermal Drug Delivery Systems. From patches to microneedles. J Drug Discov Health Sci. 2024;1(02):105–112.

[13] Yang T, Huang D, Li C, Zhao D, Li J, Zhang M, Chen Y, Wang Q, Liang Z, Liang X. Rolling microneedle electrode array (RoMEA) empowered nucleic acid delivery and cancer immunotherapy. Nano Today. 2021;36:101017.

[14] Taddio A, Chambers CT, Halperin SA, Ipp M, Lockett D, Rieder MJ, Shah V. Inadequate pain management during routine childhood immunizations: The nerve of it. Clin Ther. 2009;31:S152–S167. 19781434 10.1016/j.clinthera.2009.07.022

[15] Miao L, Zhang Y, Huang L. mRNA vaccine for cancer immunotherapy. Mol Cancer. 2021;20(1):41. 33632261 10.1186/s12943-021-01335-5 PMC7905014

[16] He X, Sun J, Zhuang J, Xu H, Liu Y, Wu D. Microneedle system for transdermal drug and vaccine delivery: Devices, safety, and prospects. Dose-Response. 2019;17(4):711639879. 10.1177/1559325819878585 PMC6794664 31662709

[17] Wang H, Xu J, Xiang L. Microneedle-mediated transcutaneous immunization: Potential in nucleic acid vaccination. Adv Healthc Mater. 2023;12(23):2300339. 10.1002/adhm.202300339 37115817

[18] Sheng T, Luo B, Zhang W, Ge X, Yu J, Zhang Y, Gu Z. Microneedle-mediated vaccination: Innovation and translation. Adv Drug Deliver Rev. 2021;179:113919. 10.1016/j.addr.2021.113919 34375682

[19] Chen S, Huang X, Xue Y, Álvarez-Benedicto E, Shi Y, Chen W, Koo S, Siegwart DJ, Dong Y, Tao W. Nanotechnology-based mRNA vaccines. Nat Rev Methods Primers. 2023;3(1):63. 40747084 10.1038/s43586-023-00246-7 PMC12312698

[20] Kim E, Erdos G, Huang S, Kenniston TW, Balmert SC, Carey CD, Raj VS, Epperly MW, Klimstra WB, Haagmans BL Microneedle array delivered recombinant coronavirus vaccines: Immunogenicity and rapid translational development. *Ebiomedicine*; 2020;55:102743. 10.1016/j.ebiom.2020.102743 PMC7128973 32249203

[21] Kuwentrai C, Yu J, Rong L, Zhang BZ, Hu YF, Gong HR, Dou Y, Deng J, Huang JD, Xu C. Intradermal delivery of receptor-binding domain of SARS-CoV-2 spike protein with dissolvable microneedles to induce humoral and cellular responses in mice. Bioeng Transl Med. 2021;6(1): Article e10202. 33349797 10.1002/btm2.10202 PMC7744900

[22] Wang L, Yang L, Zhang F, Liu X, Xie Q, Liu Q, Yuan L, Zhao T, Xie S, Xu Q. A microneedle-based delivery system for broad-protection seasonal influenza a DNA nanovaccines. Cell Rep Phys Sci. 2023;4(6).

[23] Yin Y, Su W, Zhang J, Huang W, Li X, Ma H, Tan M, Song H, Cao G, Yu S. Separable microneedle patch to protect and deliver DNA nanovaccines against COVID-19. ACS Nano. 2021;15(9):14347–14359. 34472328 10.1021/acsnano.1c03252

[24] Li M, Yang L, Wang C, Cui M, Wen Z, Liao Z, Han Z, Zhao Y, Lang B, Chen H, et al. Rapid induction of long-lasting systemic and mucosal immunity via thermostable microneedle-mediated chitosan oligosaccharide-encapsulated DNA nanoparticles. ACS Nano. 2023;17(23):24200–24217. 37991848 10.1021/acsnano.3c09521

[25] Damase TR, Sukhovershin R, Boada C, Taraballi F, Pettigrew RI, Cooke JP. The limitless future of RNA therapeutics. Front Bioeng Biotech. 2021;9:628137. 10.3389/fbioe.2021.628137 PMC8012680 33816449

[26] Yu J, Kuwentrai C, Gong H, Li R, Zhang B, Lin X, Wang X, Huang J, Xu C. Intradermal delivery of mRNA using cryomicroneedles. Acta Biomater. 2022;148:133–141. 35697200 10.1016/j.actbio.2022.06.015

[27] de Pinho Favaro MT, Atienza-Garriga J, Martínez-Torró C, Parladé E, Vázquez E, Corchero JL, Ferrer-Miralles N, Villaverde A. Recombinant vaccines in 2022: A perspective from the cell factory. Microb Cell Factories. 2022;21(1):203. 10.1186/s12934-022-01929-8 PMC9532831 36199085

[28] Gupta S, Pellett S. Recent developments in vaccine design: From live vaccines to recombinant toxin vaccines. Toxins. 2023;15(9):563. 37755989 10.3390/toxins15090563 PMC10536331

[29] Boopathy AV, Mandal A, Kulp DW, Menis S, Bennett NR, Watkins HC, Wang W, Martin JT, Thai NT, He Y. Enhancing humoral immunity via sustained-release implantable microneedle patch vaccination. Proc Natl Acad Sci. 2019;116(33):16473–16478. 31358641 10.1073/pnas.1902179116 PMC6697788

[30] Yenkoidiok-Douti L, Barillas-Mury C, Jewell CM. Design of dissolvable microneedles for delivery of a Pfs47-based malaria transmission-blocking vaccine. ACS Biomater Sci Eng. 2021;7(5):1854–1862. 33616392 10.1021/acsbiomaterials.0c01363 PMC8113916

[31] Jeong H, Bae J, Park J, Baek S, Kim G, Park M, Park J. Preclinical study of influenza bivalent vaccine delivered with a two compartmental microneedle array. J Control Release. 2020;324:280–288. 32439360 10.1016/j.jconrel.2020.05.024

[32] Schepens B, Vos PJ, Saelens X, van der Maaden K. Vaccination with influenza hemagglutinin-loaded ceramic nanoporous microneedle arrays induces protective immune responses. Eur J Pharm Biopharm. 2019;136:259–266. 30731115 10.1016/j.ejpb.2019.02.002

[33] Tran KT, Gavitt TD, Farrell NJ, Curry EJ, Mara AB, Patel A, Brown L, Kilpatrick S, Piotrowska R, Mishra N. Transdermal microneedles for the programmable burst release of multiple vaccine payloads. Nat Biomed Eng. 2021;5(9):998–1007. 33230304 10.1038/s41551-020-00650-4

[34] Wong MC, Jiang JY, Goggins WB, Liang M, Fang Y, Fung FD, Leung C, Wang HH, Wong GL, Wong VW. International incidence and mortality trends of liver cancer: A global profile. Sci Rep. 2017;7(1):45846. 28361988 10.1038/srep45846 PMC5374459

[35] Bray F, Laversanne M, Sung H, Ferlay J, Siegel RL, Soerjomataram I, Jemal A. Global cancer statistics 2022: GLOBOCAN estimates of incidence and mortality worldwide for 36 cancers in 185 countries. CA Cancer J Clin. 2024;74(3):229–263. 38572751 10.3322/caac.21834

[36] Han B, Zheng R, Zeng H, Wang S, Sun K, Chen R, Li L, Wei W, He J. Cancer incidence and mortality in China, 2022. J Natl Cancer Cent. 2024;4(1):47–53. 39036382 10.1016/j.jncc.2024.01.006 PMC11256708

[37] Ferlay J, Colombet M, Soerjomataram I, Parkin DM, Piñeros M, Znaor A, Bray F. Cancer statistics for the year 2020: An overview. Int J Cancer. 2021; 10.1002/ijc.33588. 10.1002/ijc.33588 33818764

[38] Kelly PN. The cancer immunotherapy revolution. Science. 2018;359(6382):1344–1345. 29567702 10.1126/science.359.6382.1344

[39] Klevorn LE, Teague RM. Adapting cancer immunotherapy models for the real world. Trends Immunol. 2016;37(6):354–363. 27105824 10.1016/j.it.2016.03.010 PMC4885780

[40] Zhou R, Yu H, Sheng T, Wu Y, Chen Y, You J, Yang Y, Luo B, Zhao S, Zheng Y. Grooved microneedle patch augments adoptive T cell therapy against solid tumors via diverting regulatory T cells. Adv Mater. 2024;36(30):2401667. 10.1002/adma.202401667 38843541

[41] Papadakis M, Karniadakis I, Mazonakis N, Akinosoglou K, Tsioutis C, Spernovasilis N. Immune checkpoint inhibitors and infection: What is the interplay? In Vivo. 2023;37(6):2409–2420. 37905657 10.21873/invivo.13346 PMC10621463

[42] Thummalapalli R, Ricciuti B, Bandlamudi C, Muldoon D, Rizvi H, Elkrief A, Luo J, Alessi JV, Pecci F, Lamberti G. Clinical and molecular features of long-term response to immune checkpoint inhibitors in patients with advanced non–small cell lung cancer. Clin Cancer Res. 2023;29(21):4408–4418. 37432985 10.1158/1078-0432.CCR-23-1207 PMC10618656

[43] Topalian SL, Hodi FS, Brahmer JR, Gettinger SN, Smith DC, McDermott DF, Powderly JD, Carvajal RD, Sosman JA, Atkins MB. Safety, activity, and immune correlates of anti–PD-1 antibody in cancer. New Engl J Med. 2012;366(26):2443–2454. 22658127 10.1056/NEJMoa1200690 PMC3544539

[44] Wang C, Ye Y, Hochu GM, Sadeghifar H, Gu Z. Enhanced cancer immunotherapy by microneedle patch-assisted delivery of anti-PD1 antibody. Nano Lett. 2016;16(4):2334–2340. 26999507 10.1021/acs.nanolett.5b05030

[45] Ye Y, Wang J, Hu Q, Hochu GM, Xin H, Wang C, Gu Z. Synergistic transcutaneous immunotherapy enhances antitumor immune responses through delivery of checkpoint inhibitors. ACS Nano. 2016;10(9):8956–8963. 27599066 10.1021/acsnano.6b04989

[46] Yang P, Lu C, Qin W, Chen M, Quan G, Liu H, Wang L, Bai X, Pan X, Wu C. Construction of a core-shell microneedle system to achieve targeted co-delivery of checkpoint inhibitors for melanoma immunotherapy. Acta Biomater. 2020;104:147–157. 31904558 10.1016/j.actbio.2019.12.037

[47] Chen S, Ma M, Xue F, Shen S, Chen Q, Kuang Y, Liang K, Wang X, Chen H. Construction of microneedle-assisted co-delivery platform and its combining photodynamic/immunotherapy. J Control Release. 2020;324:218–227. 32387551 10.1016/j.jconrel.2020.05.006

[48] Joo SH, Kim J, Hong J, Fakhraei Lahiji S, Kim YH. Dissolvable self-locking microneedle patches integrated with immunomodulators for cancer immunotherapy. Adv Mater. 2023;35(10):2209966. 10.1002/adma.202209966 36528846

[49] Papukashvili D, Rcheulishvili N, Liu C, Wang X, He Y, Wang PG. Strategy of developing nucleic acid-based universal monkeypox vaccine candidates. Front Immunol. 2022;13:1050309. 36389680 10.3389/fimmu.2022.1050309 PMC9646902

[50] Li Z, He Y, Deng L, Zhang Z, Lin Y. A fast-dissolving microneedle array loaded with chitosan nanoparticles to evoke systemic immune responses in mice. J Mater Chem B. 2020;8(2):216–225. 31803892 10.1039/c9tb02061f

[51] Duong HTT, Yin Y, Thambi T, Nguyen TL, Phan VG, Lee MS, Lee JE, Kim J, Jeong JH, Lee DS. Smart vaccine delivery based on microneedle arrays decorated with ultra-pH-responsive copolymers for cancer immunotherapy. Biomaterials. 2018;185:13–24. 30216806 10.1016/j.biomaterials.2018.09.008

[52] Duong HTT, Yin Y, Thambi T, Kim BS, Jeong JH, Lee DS. Highly potent intradermal vaccination by an array of dissolving microneedle polypeptide cocktails for cancer immunotherapy. J Mater Chem B. 2020;8(6):1171–1181. 31957761 10.1039/c9tb02175b

[53] Cole G, Ali AA, McCrudden CM, McBride JW, McCaffrey J, Robson T, Kett VL, Dunne NJ, Donnelly RF, McCarthy HO. DNA vaccination for cervical cancer: Strategic optimisation of RALA mediated gene delivery from a biodegradable microneedle system. Eur J Pharm Biopharm. 2018;127:288–297. 29510205 10.1016/j.ejpb.2018.02.029

[54] van der Maaden K, Heuts J, Camps M, Pontier M, Terwisscha Van Scheltinga A, Jiskoot W, Ossendorp F, Bouwstra J. Hollow microneedle-mediated micro-injections of a liposomal HPV E7(43-63) synthetic long peptide vaccine for efficient induction of cytotoxic and T-helper responses. J Control Release. 2018;269:347–354. 29174441 10.1016/j.jconrel.2017.11.035

[55] Quemener AM, Centomo ML, Sax SL, Panella R. Small drugs, huge impact: The extraordinary impact of antisense oligonucleotides in research and drug development. Molecules. 2022;27(2):536. 35056851 10.3390/molecules27020536 PMC8781596

[56] Santos R, Ursu O, Gaulton A, Bento AP, Donadi RS, Bologa CG, Karlsson A, Al-Lazikani B, Hersey A, Oprea TI. A comprehensive map of molecular drug targets. Nat Rev Drug Discov. 2017;16(1):19–34. 27910877 10.1038/nrd.2016.230 PMC6314433

[57] Yu A, Choi YH, Tu M. RNA drugs and RNA targets for small molecules: Principles, progress, and challenges. Pharmacol Rev. 2020;72(4):862–898. 32929000 10.1124/pr.120.019554 PMC7495341

[58] Li X, Xu Q, Zhang P, Zhao X, Wang Y. Cutaneous microenvironment responsive microneedle patch for rapid gene release to treat subdermal tumor. J Control Release. 2019;314:72–80. 31629710 10.1016/j.jconrel.2019.10.016

[59] Pandey PR, Young KH, Kumar D, Jain N. RNA-mediated immunotherapy regulating tumor immune microenvironment: Next wave of cancer therapeutics. Mol Cancer. 2022;21(1):58. 35189921 10.1186/s12943-022-01528-6 PMC8860277

[60] Pan J, Ruan W, Qin M, Long Y, Wan T, Yu K, Zhai Y, Wu C, Xu Y. Intradermal delivery of STAT3 siRNA to treat melanoma via dissolving microneedles. Sci Rep. 2018;8(1):1117. 29348670 10.1038/s41598-018-19463-2 PMC5773564

[61] Roth GA, Mensah GA, Johnson CO, Addolorato G, Ammirati E, Baddour LM, Barengo NC, Beaton AZ, Benjamin EJ, Benziger CP. Global burden of cardiovascular diseases and risk factors, 1990–2019: Update from the GBD 2019 study. J Am Coll Cardiol. 2020;76(25):2982–3021. 33309175 10.1016/j.jacc.2020.11.010 PMC7755038

[62] McClellan M, Brown N, Califf RM, Warner JJ. Call to action: Urgent challenges in cardiovascular disease: A presidential advisory from the American Heart Association. Circulation. 2019;139(9):e44–e54. 30674212 10.1161/CIR.0000000000000652

[63] Netala VR, Teertam SK, Li H, Zhang Z. A comprehensive review of cardiovascular disease management: Cardiac biomarkers, imaging modalities, pharmacotherapy, surgical interventions, and herbal remedies. Cells. 2024;13(17):1471. 39273041 10.3390/cells13171471 PMC11394358

[64] Saleh M, Ambrose JA. Understanding myocardial infarction. F1000Res. 2018;7: 10.12688/f1000research.15096.1. 10.12688/f1000research.15096.1 PMC6124376 30228871

[65] Chong B, Jayabaskaran J, Jauhari SM, Chan SP, Goh R, Kueh MTW, Li H, Chin YH, Kong G, Anand VV. Global burden of cardiovascular diseases: Projections from 2025 to 2050. Eur J Prev Cardiol. 2024;10.1093/eurjpc/zwae281;zwae281. 10.1093/eurjpc/zwae281 39270739

[66] Tariq U, Gupta M, Pathak S, Patil R, Dohare A, Misra SK. Role of biomaterials in cardiac repair and regeneration: Therapeutic intervention for myocardial infarction. ACS Biomater Sci Eng. 2022;8(8):3271–3298. 35867701 10.1021/acsbiomaterials.2c00454

[67] White SJ, Chong JJH. Growth factor therapy for cardiac repair: An overview of recent advances and future directions. Biophys Rev. 2020;12(4):805–815. 32691300 10.1007/s12551-020-00734-0 PMC7429584

[68] Li Z, Hu S, Cheng K. Chemical engineering of cell therapy for heart diseases. Acc Chem Res. 2019;52(6):1687–1696. 31125198 10.1021/acs.accounts.9b00137 PMC7045701

[69] Hu S, Zhu D, Li Z, Cheng K. Detachable microneedle patches deliver mesenchymal stromal cell factor-loaded nanoparticles for cardiac repair. ACS Nano. 2022;16(10):15935–15945. 36148975 10.1021/acsnano.2c03060

[70] Fan Z, Wei Y, Yin Z, Huang H, Liao X, Sun L, Liu B, Liu F. Near-infrared light-triggered unfolding microneedle patch for minimally invasive treatment of myocardial ischemia. Acs Appl Mater Inter. 2021;13(34):40278–40289. 10.1021/acsami.1c09658 34424666

[71] Yang Q, Fang J, Lei Z, Sluijter JP, Schiffelers R. Repairing the heart: State-of the art delivery strategies for biological therapeutics. Adv Drug Deliver Rev. 2020;160:1–18. 10.1016/j.addr.2020.10.003 33039498

[72] Lim S, Park TY, Jeon EY, Joo KI, Cha HJ. Double-layered adhesive microneedle bandage based on biofunctionalized mussel protein for cardiac tissue regeneration. Biomaterials. 2021;278: Article 121171. 34624751 10.1016/j.biomaterials.2021.121171

[73] Vale PR, Losordo DW, Tkebuchava T, Chen D, Milliken CE, Isner JM. Catheter-based myocardial gene transfer utilizing nonfluoroscopic electromechanical left ventricular mapping. J Am Coll Cardiol. 1999;34(1):246–254. 10400018 10.1016/s0735-1097(99)00143-6

[74] Shi H, Xue T, Yang Y, Jiang C, Huang S, Yang Q, Lei D, You Z, Jin T, Wu F. Microneedle-mediated gene delivery for the treatment of ischemic myocardial disease. Sci Adv. 2020;6(25): Article eaaz3621. 32596444 10.1126/sciadv.aaz3621 PMC7299628

[75] Gao F, Kataoka M, Liu N, Liang T, Huang Z, Gu F, Ding J, Liu J, Zhang F, Ma Q. Therapeutic role of miR-19a/19b in cardiac regeneration and protection from myocardial infarction. Nat Commun. 2019;10(1):1802. 30996254 10.1038/s41467-019-09530-1 PMC6470165

[76] Li B, Hu J, Chen X. MicroRNA-30b protects myocardial cell function in patients with acute myocardial ischemia by targeting plasminogen activator inhibitor-1. Exp Ther Med. 2018;15(6):5125–5132. 29805539 10.3892/etm.2018.6039 PMC5958726

[77] Chen X, Chen H, Zhu L, Zeng M, Wang T, Su C, Vulugundam G, Gokulnath P, Li G, Wang X. Nanoparticle–patch system for localized, effective, and sustained miRNA administration into infarcted myocardium to alleviate myocardial ischemia–reperfusion injury. ACS Nano. 2024;18(30):19470–19488. 10.1021/acsnano.3c08811 39020456

[78] Melo SF, Fernandes T, Baraúna VG, Matos KC, Santos AA, Tucci PJ, Oliveira EM. Expression of microRNA-29 and collagen in cardiac muscle after swimming training in myocardial-infarcted rats. Cell Physiol Biochem. 2014;33(3):657–669. 24642957 10.1159/000358642

[79] Yuan J, Yang H, Liu C, Shao L, Zhang H, Lu K, Wang J, Wang Y, Yu Q, Zhang Y. Microneedle patch loaded with exosomes containing microRNA-29b prevents cardiac fibrosis after myocardial infarction. Adv Healthc Mater. 2023;12(13):2202959. 10.1002/adhm.202202959 36739582

[80] Raskob GE, Angchaisuksiri P, Blanco AN, Buller H, Gallus A, Hunt BJ, Hylek EM, Kakkar A, Konstantinides SV, McCumber M. Thrombosis: A major contributor to global disease burden. Arterioscler Thromb Vasc Biol. 2014;34(11):2363–2371. 25304324 10.1161/ATVBAHA.114.304488

[81] Bruni-Fitzgerald KR. Venous thromboembolism: An overview. J Vasc Nurs. 2015;33(3):95–99. 26298612 10.1016/j.jvn.2015.02.001

[82] Arshad MS, Zafar S, Zahra AT, Zaman MH, Akhtar A, Kucuk I, Farhan M, Chang M, Ahmad Z. Fabrication and characterisation of self-applicating heparin sodium microneedle patches. J Drug Target. 2021;29(1):60–68. 32649227 10.1080/1061186X.2020.1795180

[83] Zhang Y, Yu J, Wang J, Hanne NJ, Cui Z, Qian C, Wang C, Xin H, Cole JH, Gallippi CM. Thrombin-responsive transcutaneous patch for auto-anticoagulant regulation. Adv Mater. 2017;29(4): 10.1002/adma.201604043. 10.1002/adma.201604043 PMC5250559 27885722

[84] Zehnder JL. Drugs used in disorders of coagulation. Basic Clin Pharmacol. 2018;11:587–599.

[85] Lichota A, Szewczyk EM, Gwozdzinski K. Factors affecting the formation and treatment of thrombosis by natural and synthetic compounds. Int J Mol Sci. 2020;21(21):7975. 33121005 10.3390/ijms21217975 PMC7663413

[86] Junren C, Xiaofang X, Huiqiong Z, Gangmin L, Yanpeng Y, Xiaoyu C, Yuqing G, Yanan L, Yue Z, Fu P. Pharmacological activities and mechanisms of hirudin and its derivatives—A review. Front Pharmacol. 2021;12: Article 660757. 33935784 10.3389/fphar.2021.660757 PMC8085555

[87] Men Z, Lu X, He T, Wu M, Su T, Shen T. Microneedle patch-assisted transdermal administration of recombinant hirudin for the treatment of thrombotic diseases. Int J Pharmaceut. 2022;612: Article 121332. 10.1016/j.ijpharm.2021.121332 34902453

[88] Blondin C, de Agostiniz A. Biological activities of polysaccharides from marine algae. Drugs Fut. 1995;20:1237–1249.

[89] Stephanie S, Enggi CK, Sulistiawati S, Tangdilintin F, Achmad AA, Litaay M, Kleuser B, Manggau MA, Permana AD. Fucoidan-incorporated dissolving microneedles: A novel approach to anticoagulant transdermal delivery. J Drug Deliv Sci Tec. 2024;95: Article 105587.

[90] Neeland IJ, Lim S, Tchernof A, Gastaldelli A, Rangaswami J, Ndumele CE, Powell-Wiley TM, Després J. Metabolic syndrome. Nat Rev Dis Primers. 2024;10(1):77. 39420195 10.1038/s41572-024-00563-5

[91] Dou Q, Chakrabarty S, Wu D, Tam L, Jin YZ, So H, Cerin E, Barnett A, Mubarik S, Hezam KA. The temporal trend of disease burden attributable to metabolic risk factors in China: An analysis of the global burden of disease study. *Nutr Metab Aging*. 2023. 10.3389/fnut.2022.1035439 PMC9846330 36687675

[92] Wu Y, Lin Z, Li C, Lin X, Shan S, Guo B, Zheng M, Li F, Yuan L, Li Z. Epigenetic regulation in metabolic diseases: Mechanisms and advances in clinical study. Signal Transduct Target Ther. 2023;8(1):98. 36864020 10.1038/s41392-023-01333-7 PMC9981733

[93] Ong KL, Stafford LK, McLaughlin SA, Boyko EJ, Vollset SE, Smith AE, Dalton BE, Duprey J, Cruz JA, Hagins H. Global, regional, and national burden of diabetes from 1990 to 2021, with projections of prevalence to 2050: A systematic analysis for the global burden of disease study 2021. Lancet. 2023;402(10397):203–234. 37356446 10.1016/S0140-6736(23)01301-6 PMC10364581

[94] Mathieu C, Martens P, Vangoitsenhoven R. One hundred years of insulin therapy. Nat Rev Endocrinol. 2021;17(12):715–725. 34404937 10.1038/s41574-021-00542-w

[95] Zong Q, Guo R, Dong N, Ling G, Zhang P. Design and development of insulin microneedles for diabetes treatment. Drug Deliv Transl Re. 2021;12(5):973–980. 10.1007/s13346-021-00981-y 33851362

[96] Yu J, Zhang Y, Ye Y, DiSanto R, Sun W, Ranson D, Ligler FS, Buse JB, Gu Z. Microneedle-array patches loaded with hypoxia-sensitive vesicles provide fast glucose-responsive insulin delivery. Proc Natl Acad Sci USA. 2015;112(27):8260–8265. 26100900 10.1073/pnas.1505405112 PMC4500284

[97] Chen X, Dou X, Qiu W. Promising strategies for smart insulin delivery system: Glucose-sensitive microneedle. Eur J Med Chem. 2024;278: Article 116793. 39216380 10.1016/j.ejmech.2024.116793

[98] Yang D, Cai C, Liu K, Peng Z, Yan C, Xi J, Xie F, Li X. Recent advances in glucose-oxidase-based nanocomposites for diabetes diagnosis and treatment. J Mater Chem B. 2023;11(32): 10.1039/d3tb01097j. 10.1039/d3tb01097j 37522237

[99] Chen Q, Xiao Z, Wang C, Chen G, Zhang Y, Zhang X, Han X, Wang J, Ye X, Prausnitz MR, et al. Microneedle patches loaded with nanovesicles for glucose transporter-mediated insulin delivery. ACS Nano. 2022;16(11):18223–18231. 36322923 10.1021/acsnano.2c05687 PMC10738036

[100] Yu J, Wang J, Zhang Y, Chen G, Mao W, Ye Y, Kahkoska AR, Buse JB, Langer R, Gu Z. Glucose-responsive insulin patch for the regulation of blood glucose in mice and minipigs. Nat Biomed Eng. 2020;4(5):499–506. 32015407 10.1038/s41551-019-0508-y PMC7231631

[101] Zong Q, Zhou R, Zhao Z, Wang Y, Liu C, Zhang P. Glucose-responsive insulin microneedle patch based on phenylboronic acid for 1 diabetes treatment. Eur Polym J. 2022;173: Article 111217.

[102] Liu JF, GhavamiNejad A, Lu B, Mirzaie S, Samarikhalaj M, Giacca A, Wu XY. “Smart” matrix microneedle patch made of self-crosslinkable and multifunctional polymers for delivering insulin on-demand. Adv Sci. 2023;10(30): Article e2303665. 10.1002/advs.202303665 PMC10602565 37718654

[103] Yu J. Bioresponsive patches for transdermal drug delivery [dissertation]. North Carolina State University; 2018.

[104] Kim S, Yang H, Eum J, Ma Y, Lahiji SF, Jung H. Implantable powder-carrying microneedles for transdermal delivery of high-dose insulin with enhanced activity. Biomaterials. 2020;232: Article 119733. 31901501 10.1016/j.biomaterials.2019.119733

[105] Zhang X, Chen G, Cai L, Fan L, Zhao Y. Dip-printed microneedle motors for oral macromolecule delivery. Research. 2022;2022: Article 9797482. 35958112 10.34133/2022/9797482 PMC9343079

[106] Zhao X, Wang M, Wen Z, Lu Z, Cui L, Fu C, Xue H, Liu Y, Zhang Y. GLP-1 receptor agonists: Beyond their pancreatic effects. Front Endocrinol. 2021;12: Article 721135. 10.3389/fendo.2021.721135 PMC8419463 34497589

[107] You J, Yang C, Han J, Wang H, Zhang W, Zhang Y, Lu Z, Wang S, Cai R, Li H. Ultrarapid-acting microneedles for immediate delivery of biotherapeutics. Adv Mater. 2023;35(45):2304582. 10.1002/adma.202304582 37547966

[108] Chen W, Tian R, Xu C, Yung BC, Wang G, Liu Y, Ni Q, Zhang F, Zhou Z, Wang J, et al. Microneedle-array patches loaded with dual mineralized protein/peptide particles for type 2 diabetes therapy. Nat Commun. 2017;8(1):1777. 29176623 10.1038/s41467-017-01764-1 PMC5701150

[109] You J, Juhng S, Song J, Park J, Jang M, Kang G, Yang H, Min HS, Shin J, Lee S. Egg microneedle for transdermal delivery of active liraglutide. Adv Healthc Mater. 2023;12(9):2202473. 10.1002/adhm.202202473 36617627

[110] Chen W, Wainer J, Ryoo SW, Qi X, Chang R, Li J, Lee SH, Min S, Wentworth A, Collins JE. Dynamic omnidirectional adhesive microneedle system for oral macromolecular drug delivery. Sci Adv. 2022;8(1): Article eabk1792. 34985942 10.1126/sciadv.abk1792 PMC8730401

[111] David P, Singh S, Ankar R. A comprehensive overview of skin complications in diabetes and their prevention. Cureus J Med Sci. 2023;15(5): Article e38961. 10.7759/cureus.38961 PMC10259731 37313065

[112] Rodríguez-Rodríguez N, Martínez-Jiménez I, García-Ojalvo A, Mendoza-Mari Y, Guillén-Nieto G, Armstrong DG, Berlanga-Acosta J. Wound chronicity, impaired immunity and infection in diabetic patients. MEDICC Rev. 2022;24:44–58. 34653116 10.37757/MR2021.V23.N3.8

[113] Zhang X, Wang Z, Jiang H, Zeng H, An N, Liu B, Sun L, Fan Z. Self-powered enzyme-linked microneedle patch for scar-prevention healing of diabetic wounds. Sci Adv. 2023;9(28): Article eadh1415. 37450590 10.1126/sciadv.adh1415 PMC10348682

[114] Chooi YC, Ding C, Magkos F. The epidemiology of obesity. Metabolism. 2019;92:6–10. 30253139 10.1016/j.metabol.2018.09.005

[115] Wolin KY, Carson K, Colditz GA. Obesity and cancer. Oncologist. 2010;15(6):556–565. 20507889 10.1634/theoncologist.2009-0285 PMC3227989

[116] Wang J, Wang Q, Yang X, Yang W, Li D, Jin J, Zhang H, Zhang X. GLP-1 receptor agonists for the treatment of obesity: Role as a promising approach. Front Endocrinol. 2023;14:1085799. 10.3389/fendo.2023.1085799 PMC9945324 36843578

[117] Singh P, Vinikoor T, Sharma N, Nelson N, Prasadh S, Oiknine R, Nguyen TD. Single-administration self-boosting microneedle patch for the treatment of obesity. Adv Ther-Germany. 2024;7(9):2400028. 10.1002/adtp.202400028 PMC11486425 39429250

[118] Juhng S, Song J, You J, Park J, Yang H, Jang M, Kang G, Shin J, Ko HW, Jung H. Fabrication of liraglutide-encapsulated triple layer hyaluronic acid microneedles(TLMs) for the treatment of obesity. Lab Chip. 2023;23(10):2378–2388. 36919574 10.1039/d2lc01084d

[119] Jalleh RJ, Rayner CK, Hausken T, Jones KL, Camilleri M, Horowitz M. Gastrointestinal effects of GLP-1 receptor agonists: Mechanisms, management, and future directions. Lancet Gastroenterol Hepatol. 2024;9(10):957–964. 39096914 10.1016/S2468-1253(24)00188-2

[120] Angelidi AM, Belanger MJ, Kokkinos A, Koliaki CC, Mantzoros CS. Novel noninvasive approaches to the treatment of obesity: From pharmacotherapy to gene therapy. Endocr Rev. 2022;43(3):507–557. 35552683 10.1210/endrev/bnab034 PMC9113190

[121] Chung JY, Ain QU, Song Y, Yong S, Kim Y. Targeted delivery of CRISPR interference system against Fabp4 to white adipocytes ameliorates obesity, inflammation, hepatic steatosis, and insulin resistance. Genome Res. 2019;29(9):1442–1452. 31467027 10.1101/gr.246900.118 PMC6724665

[122] Chung JY, Hong J, Kim H, Song Y, Yong S, Lee J, Kim Y. White adipocyte-targeted dual gene silencing of FABP4/5 for anti-obesity, anti-inflammation and reversal of insulin resistance: Efficacy and comparison of administration routes. Biomaterials. 2021;279: Article 121209. 34700224 10.1016/j.biomaterials.2021.121209

[123] Choi H, Hong J, Seo Y, Joo SH, Lim H, Lahiji SF, Kim YH. Self-assembled oligopeptoplex-loaded dissolving microneedles for adipocyte-targeted anti-obesity gene therapy. Adv Mater. 2024;36(16):2309920. 10.1002/adma.202309920 38213134

[124] Rossini M, Adami S, Bertoldo F, Diacinti D, Gatti D, Giannini S, Giusti A, Malavolta N, Minisola S, Osella G. Guidelines for the diagnosis, prevention and management of osteoporosis. Reumatismo. 2016;68(1):1–39. 27339372 10.4081/reumatismo.2016.870

[125] Song S, Guo Y, Yang Y, Fu D. Advances in pathogenesis and therapeutic strategies for osteoporosis. Pharmacol Therapeut. 2022;237: Article 108168. 10.1016/j.pharmthera.2022.108168 35283172

[126] Marcus R, Dempster DW, Cauley JA, Feldman D. *Osteoporosis*. Academic Press; 2013.

[127] Srinivasan A, Wong FK, Karponis D. Calcitonin: A useful old friend. J Musculoskel Neuron. 2020;20(4):600. PMC7716677 33265089

[128] Tas C, Mansoor S, Kalluri H, Zarnitsyn VG, Choi S, Banga AK, Prausnitz MR. Delivery of salmon calcitonin using a microneedle patch. Int J Pharmaceut. 2012;423(2):257–263. 10.1016/j.ijpharm.2011.11.046 PMC3273586 22172290

[129] Li Y, Ju X, Fu H, Zhou C, Gao Y, Wang J, Xie R, Wang W, Liu Z, Chu L. Composite separable microneedles for transdermal delivery and controlled release of salmon calcitonin for osteoporosis therapy. ACS Appl Mater Inter. 2022;15(1):638–650. 10.1021/acsami.2c19241 36576723

[130] Mo X, Meng K, Li Z, Lan S, Ren Z, Fu X, Li C, Sun T, Xie D, Zhang Z, et al. An integrated microcurrent delivery system facilitates human parathyroid hormone delivery for enhancing osteoanabolic effect. Small Methods. 2024;3: Article e2401144. 10.1002/smtd.202401144 PMC11926516 39420694

[131] Sim J, Kang G, Yang H, Jang M, Kim Y, Ahn H, Kim M, Jung H. Development of clinical weekly-dose teriparatide acetate encapsulated dissolving microneedle patch for efficient treatment of osteoporosis. Polymers-Basel. 2022;14(19): Article 4027. 36235975 10.3390/polym14194027 PMC9571303

[132] Mezgebu E, Aliy A, Worku T. Autoimmunity and immune tolerance: A review. Microbiol Res Intl. 2023;11(2):23–35.

[133] Zhang F, Cui Y, Gao X. Time trends in the burden of autoimmune diseases across the BRICS: An age-period-cohort analysis for the GBD 2019. RMD Open. 2023;9: Article e003650. 38056916 10.1136/rmdopen-2023-003650 PMC10711932

[134] Conrad N, Verbeke G, Molenberghs G, Goetschalckx L, Callender T, Cambridge G, Mason JC, Rahimi K, McMurray JJ, Verbakel JY. Autoimmune diseases and cardiovascular risk: A population-based study on 19 autoimmune diseases and 12 cardiovascular diseases in 22 million individuals in the UK. Lancet. 2022;400(10354):733–743. 36041475 10.1016/S0140-6736(22)01349-6

[135] Costa E, Machado M, Pintado M, Silva S. Biological macromolecules as immunomodulators. In: *Biological macromolecules*. Elsevier; 2022. p. 273–287.

[136] Zhao Y, Chen X, He P, Wang X, Xu Y, Hu R, Ou Y, Zhang Z, Zhang Z, Du G. Transdermal microneedles alleviated rheumatoid arthritis by inducing immune tolerance via skin-resident antigen presenting cells. Small. 2024;20(16):2307366. 10.1002/smll.202307366 38039446

[137] Radu A, Bungau SG. Management of rheumatoid arthritis: An overview. Cells. 2021;10(11):2857. 34831081 10.3390/cells10112857 PMC8616326

[138] Tanaka Y. Recent progress in treatments of rheumatoid arthritis: An overview of developments in biologics and small molecules, and remaining unmet needs. Rheumatology. 2021;60(Supplement_6):vi12–vi20. 34951925 10.1093/rheumatology/keab609 PMC8709568

[139] Du G, He P, Zhao J, He C, Jiang M, Zhang Z, Zhang Z, Sun X. Polymeric microneedle-mediated transdermal delivery of melittin for rheumatoid arthritis treatment. J Control Release. 2021;336:537–548. 34237400 10.1016/j.jconrel.2021.07.005

[140] Cao J, Zhang N, Wang Z, Su J, Yang J, Han J, Zhao Y. Microneedle-assisted transdermal delivery of etanercept for rheumatoid arthritis treatment. Pharmaceutics. 2019;11(5):–235. 10.3390/pharmaceutics11050235 PMC6572071 31096705

[141] Zhou X, Chen Y, Cui L, Shi Y, Guo C. Advances in the pathogenesis of psoriasis: From keratinocyte perspective. Cell Death Dis. 2022;13(1):81. 35075118 10.1038/s41419-022-04523-3 PMC8786887

[142] Li Z, Zhao P, Ling Z, Zheng Y, Qu F, Chang H. An Ultraswelling microneedle device for facile and efficient drug loading and transdermal delivery. Adv Healthc Mater. 2024;13(2):2302406. 10.1002/adhm.202302406 37861278

[143] Wu D, Shou X, Yu Y, Wang X, Chen G, Zhao Y, Sun L. Biologics-loaded photothermally dissolvable hyaluronic acid microneedle patch for psoriasis treatment. Adv Funct Mater. 2022;32(47):2205847.

[144] Wan T, Pan Q, Ping Y. Microneedle-assisted genome editing: A transdermal strategy of targeting NLRP3 by CRISPR-Cas9 for synergistic therapy of inflammatory skin disorders. Sci Adv. 2021;7(11): Article eabe2888. 33692106 10.1126/sciadv.abe2888 PMC7946375

[145] Leng F, Zheng M, Xu C. 3D-printed microneedles with open groove channels for liquid extraction. Exploration. 2021;1(3):20210109. 37323692 10.1002/EXP.20210109 PMC10190842

[146] Cai Y, Huang S, Zhang Z, Zhang J, Zhu X, Chen X, Ding X. Bioinspired rotation microneedles for accurate transdermal positioning and ultraminimal-invasive biomarker detection with mechanical robustness. Research. 2022;2022: Article 9869734. 35350471 10.34133/2022/9869734 PMC8924791

[147] Zhang Y, Xu Y, Kong H, Zhang J, Chan HF, Wang J, Shao D, Tao Y, Li M. Microneedle system for tissue engineering and regenerative medicine. Exploration. 2023;3(1):20210170. 37323624 10.1002/EXP.20210170 PMC10190997

[148] Arikat F, Hanna SJ, Singh RK, Vilela L, Wong FS, Dayan CM, Coulman SA, Birchall JC. Targeting proinsulin to local immune cells using an intradermal microneedle delivery system; a potential antigen-specific immunotherapy for type 1 diabetes. J Control Release. 2020;322:593–601. 32087298 10.1016/j.jconrel.2020.02.031

